# Identification of BLNK and BTK as mediators of rituximab‐induced programmed cell death by CRISPR screens in GCB‐subtype diffuse large B‐cell lymphoma

**DOI:** 10.1002/1878-0261.12753

**Published:** 2020-07-16

**Authors:** Emil Aagaard Thomsen, Anne Bruun Rovsing, Mads Valdemar Anderson, Hanne Due, Jinrong Huang, Yonglun Luo, Karen Dybkær, Jacob Giehm Mikkelsen

**Affiliations:** ^1^ Department of Biomedicine Aarhus University Denmark; ^2^ Department of Hematology Aalborg University Hospital Denmark; ^3^ Lars Bolund Institute of Regenerative Medicine BGI‐Qingdao BGI‐Shenzhen China; ^4^ Department of Biology University of Copenhagen Denmark

**Keywords:** B‐cell receptor, CD20, CRISPR, CRISPR library screen, lentiviral vectors, rituximab

## Abstract

Diffuse large B‐cell lymphoma (DLBCL) is characterized by extensive genetic heterogeneity, and this results in unpredictable responses to the current treatment, R‐CHOP, which consists of a cancer drug combination supplemented with the humanized CD20‐targeting monoclonal antibody rituximab. Despite improvements in the patient response rate through rituximab addition to the treatment plan, up to 40% of DLBCL patients end in a relapsed or refractory state due to inherent or acquired resistance to the regimen. Here, we employ a lentiviral genome‐wide clustered regularly interspaced short palindromic repeats library screening approach to identify genes involved in facilitating the rituximab response in cancerous B cells. Along with the CD20‐encoding *MS4A1* gene, we identify genes related to B‐cell receptor (BCR) signaling as mediators of the intracellular signaling response to rituximab. More specifically, the B‐cell linker protein (*BLNK*) and Bruton's tyrosine kinase (*BTK*) genes stand out as pivotal genes in facilitating direct rituximab‐induced apoptosis through mechanisms that occur alongside complement‐dependent cytotoxicity (CDC). Our findings demonstrate that rituximab triggers BCR signaling in a BLNK‐ and BTK‐dependent manner and support the existing notion that intertwined CD20 and BCR signaling pathways in germinal center B‐cell‐like‐subtype DLBCL lead to programmed cell death.

## Graphical abstract

Genome‐wide clustered regularly interspaced short palindromic repeats‐based screening **identify** B‐cell receptor (BCR) signaling as a core **mediator** of rituximab sensitivity in OCI‐Ly‐7 cells, specifically through actions of the B‐cell linker protein and Bruton's tyrosine kinase proteins. **This supports the notion of** intertwined signaling pathways in germinal center B‐cell‐like‐subtype diffuse large B‐cell lymphoma involving stimulation of CD20 and BCR on the cellular path toward programmed cell death and reduction of CD20 levels.

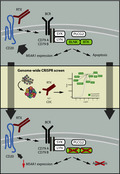

**Abbreviations**
ABC: activated B‐cell‐like
BCR: B‐cell receptor
*BLNK*: B‐cell linker protein
*BTK*: Bruton's tyrosine kinase
CDC: complement‐dependent cytotoxicity
CRISPR: clustered regularly interspaced short palindromic repeats
DLBCL: diffuse large B‐cell lymphoma
FDR: false discovery rate
GCB: germinal center B‐cell‐like
GSEA: gene set enrichment analysis
HIHS: heat‐inactivated human serum
HS: human serum
KO: knockout
MOI: multiplicity of infection
NGS: next‐generation sequencing
NHL: non‐Hodgkin lymphomas
R‐CHOP: rituximab (R), cyclophosphamide (C), doxorubicin (H), vincristine (O), prednisone (P)
RTX: rituximab
RTX‐REC: (repeated exposure to CDC conditions, REC)
RTX‐SEC: (short exposure to CDC conditions, SEC)
sgRNA: single‐guide RNA

## 1 Introduction

Diffuse large B‐cell lymphoma (DLBCL) is the predominant subtype of non‐Hodgkin lymphomas (NHL) accounting for around 30% of NHL cases [1]. DLBCL is characterized by extensive genetic and molecular heterogeneity, which results in unpredictable and varying responses to treatment [2, 3, 4, 5]. DLBCL subclasses have been distinguished by microarray‐based gene expression profiling and divided into activated B‐cell‐like (ABC) and germinal center B‐cell‐like (GCB) [6]. This classification reflects the cell of origin and distinguishes the two subclasses of DLBCL with regard to genetic alterations, oncogenic mechanisms, and clinical outcome [5, 7]. Recently, next‐generation sequencing (NGS) methods have confirmed the molecular subclassification and further contributed to the exploration of DLBCL heterogeneity, unveiling new details on genetic drivers, differences between ABC and GCB subclasses, and their impact on clinical outcome [2, 3, 4].

R‐CHOP, a cocktail of drugs consisting of rituximab (R), cyclophosphamide (C), doxorubicin (H), vincristine (O), and prednisone (P), constitutes the current standard regimen for the treatment of DLBCL. Supplementing CHOP with rituximab (RTX) has improved the response rate from 63% to 76% [8, 9], but 30–35% of patients progress to relapse or refractory disease and eventually succumb [10]. Stratification by ABC or GCB subclass differentiates the R‐CHOP response, with the 5‐year overall survival for GCB being 69 ± 3% compared to 53 ± 3% for patients with ABC‐type DLBCL [5]. RTX is a chimeric monoclonal antibody targeting CD20, a protein expressed and presented on the surface of B cells through various developmental stages [11]. Binding of RTX to CD20 induces complement‐dependent cytotoxicity (CDC) via the classic pathway [12] and/or recruitment of immune effector cells, leading to antibody‐dependent cellular cytotoxicity [13, 14]. Moreover, recognition of CD20 by RTX leads to the aggregation in lipid rafts [15], which initiates a cascade of intracellular signaling events involving calcium influx [16, 17] and phosphorylation by SRC family kinases [18, 19], leading to apoptosis and/or cell cycle arrest [18, 20, 21, 22].

Clustered regularly interspaced short palindromic repeats (CRISPR), part of the adaptive immune system in bacteria [23], has emerged as a powerful tool for introduction of knockout (KO) mutations in predetermined genomic loci in eukaryotic cells [24]. CRISPR action is based on single‐guide RNA (sgRNA)‐guided recruitment of Cas9 endonuclease to a genomic locus, leading to formation of a targeted double‐stranded DNA break [25] and insertion or deletion of one or more base pairs after repair by nonhomologous end joining. CRISPR‐based gene KO is the hallmark of genome‐wide screening technologies based on lentiviral delivery of sgRNAs targeting all genes, allowing identification of genes affecting a specific cellular phenotype of interest. CRISPR screens have successfully identified genes driving cell proliferation and tumor growth in various cancers [26, 27] and were utilized to characterize functional drivers and unravel interactions of ibrutinib in DLBCL [3, 28]. Here, we exploit the power of genome‐wide CRISPR screens to identify genes affecting the resistance of cancerous B cells to RTX *via* complement‐dependent mechanisms and direct depletion. Our studies reveal that KO mutations in only one single gene, the *MS4A1* gene encoding CD20, are able to rescue cell depletion induced by CDC. Furthermore, our findings support a central role of B‐cell receptor (BCR) signaling in facilitating direct RTX‐induced apoptosis in the absence of CDC and point to the Bruton's tyrosine kinase (BTK) and B‐cell linker protein (BLNK) proteins as key mediators of sensitivity to direct RTX‐induced cell depletion in GCB‐DLBCL.

## 2 Materials and methods

### 2.1 Cell lines

HEK293T and the DLBCL cell lines OCI‐Ly‐7, SU‐DHL‐5, and RIVA were maintained as previously described [29].

### 2.2 Plasmid construction

pLentiCRISPR v2 (Addgene plasmid # 52961; http://n2t.net/addgene:52961; RRID:Addgene_52961) [30] and pLentiCas9‐Blast (Addgene plasmid # 52962; http://n2t.net/addgene:52962; RRID:Addgene_52962) [30] were kindly provided by Feng Zhang. pLX_311‐KRAB‐dCas9 was a gift from John Doench, William Hahn, and David Root (Addgene plasmid # 96918; http://n2t.net/addgene:96918; RRID:Addgene_96918) [31]. plentiCRISPR V2 Ctrl sgRNA was generated previously [29]. pCCL/PGK‐eGFP has been described previously [32]. sgRNA design was performed using GPP sgRNA designer from Broad Institute [33, 34], or sgRNA sequences were derived from either Human GeCKOv2 CRISPR KO pooled library or Human Brunello CRISPR KO pooled library and are available in Appendix S1. Cloning of sgRNAs into different backbones was performed as described in Ref. [35], but using Esp3I. All restriction enzymes were purchased from Thermo Fisher Scientific, Waltham, MA, USA. plentiGuide‐Puro was cloned by digesting plentiGuide‐Puro Gecko library part A with SmaI and NdeI. The sgRNA scaffold was amplified from pLentiCRISPR v2 and part of U6 amplified from pLentiGuide‐Puro Gecko library part A. Fragments were assembled using NEBuilder® HiFi DNA Assembly Master Mix (New England Biolabs, Ipswich, MA, USA). pCCL/PGK‐MS4A1 was cloned by digesting pCCL/PGK‐eGFP with BoxI and XhoI. To introduce silent mutations in the MS4A1 sgRNA 1 binding site, *MS4A1* cDNA was amplified in three separate reactions, introducing silent mutations in the overlapping regions. Fragments were assembled using NEBuilder® HiFi DNA Assembly Master Mix.

### 2.3 Lentiviral vector production and titration

Lentiviral production was performed in HEK293T cells as previously described [36]. Titration in OCI‐Ly‐7 was performed by quantification of proviral elements in genomic DNA as previously described. Transductions were performed without any additives in standard culture medium.

### 2.4 Genome‐wide CRISPR screening

OCI‐Ly‐7 cells were screened using the GeCKOv2 genome‐wide library, essentially as previously described [37], at three different conditions: (a) medium containing human serum (HS; mock), (b) medium containing 10 µg·mL^−1^ RTX (MabThera®; Roche, Copenhagen, Denmark), one‐time administration of HS [exposure to CDC conditions, SEC (RTX‐SEC); 14 days], and (c) medium with 10 µg·mL^−1^ RTX increased to 25 µg·mL^−1^ at day 16 and with repeated re‐administration of HS [repeated exposure to CDC conditions, REC (RTX‐REC); 21 days].

Human GeCKOv2 CRISPR KO pooled library was a gift from Feng Zhang (Addgene #1000000049, Watertown, MA, USA). After plasmid amplification of the library as described previously [37], intact sgRNA representation was validated by targeted NGS. Sequencing detected 99.98% of the 123 411 sgRNAs contained in the Gecko v2 library in our plasmid pool, and all protein‐encoding genes were still targeted by at least five sgRNAs. Furthermore, 98.23% of sgRNAs were covered by 20 reads or more. After lentiviral preparation of the library, screening was initiated at 1000 copies per sgRNA, following transduction [transduced at 0.5 multiplicity of infection (MOI); titer estimation in Fig. S2A]. Throughout the duration of the screen, a library coverage of 1000 copies pr. sgRNA was maintained when passaging cells. A minimum of 1.25 × 10^8^ cells were harvested per sample, and genomic DNA was purified. PCR preparations for sequencing were carried out by a nested PCR approach. The first PCR was run on 256 μg gDNA to cover the representation of sgRNAs at 500 copies at 20 cycles. Using 5′‐TGTGGAAAGGACGAAACACC‐3′ (forward) and 5′‐GTTTGTATGTCTGTTGCTAT‐3′ (reverse), thirteen individual PCR two reactions were run at 18 cycles, and the resulting 250 bp PCR amplicon was run on a gel and purified by gel extraction. Next‐generation amplicon sequencing was carried out at BGI‐Research, Shenzhen. Briefly, PCR amplicons were processed by end repair and ligated to BGISEQ sequencer compatible adapters, generating DNB‐based sequencing libraries without PCR amplification. The quality and quantity of the sequencing libraries were assessed using Agilent 2100 BioAnalyzer (Agilent Technologies, Santa Clara, CA, USA). Finally, the libraries were sequenced on the BGISEQ‐500 (MGI Tech., Shenzhen, China) with 50 paired‐end read (PE50). Mapping of sgRNA reads was performed by MAGeCK version 0.5.8 count with default parameters, following mapping sample coverage ranging from 106 to 218. After the mapping of sgRNAs, sgRNAs targeting microRNA‐encoding genes were filtered out to focus on protein‐encoding genes. Analysis was performed with MAGeCK version 0.5.8 test function using default parameters and the built‐in control sgRNAs from the Gecko v2 library. The sequencing data from our genome‐wide CRISPR screen are available at NCBI gene expression omnibus, under accession number GSE139385.

### 2.5 Gene set enrichment analysis

Gene set enrichment analysis (GSEA) was performed using Enrichr [38, 39]. A weighted value for each gene was calculated by subtracting the false discovery rate (FDR) value from 1. The genes submitted to Enrichr, the resulting top five pathways from five different databases, and the different databases used to compile the set of BCR‐related genes are available from Appendix S2.

### 2.6 Rituximab cell viability assay

All assays were performed with 20% serum added. Assays with heat‐inactivated Pooled Normal Human Male AB Serum (Innovative Research, Novy, MI, USA) had 50 µg·mL^−1^ RTX (MabThera^®^; Roche) in 1 mL total; HS was heat‐inactivated at 56 °C for 30 min. Assays with active HS had 10 µg·mL^−1^ RTX. In all assays, saline controls were included. For assays spanning 24 and 48 h, 3 × 10^5^ cells were seeded, whereas 1.5 × 10^5^ cells were seeded in 72‐h assays. Following treatment, living cells were counted using trypan blue exclusion, using a Neubauer chamber (0.0025 mm^2^). Saline‐treated populations were included to account for population‐specific growth rate in the absence of RTX.

### 2.7 Apoptosis assay

Following treatment with RTX, apoptosis levels in each population were determined by annexin staining and quantified by flow cytometry.

### 2.8 Flow cytometry

Levels of RTX‐induced apoptosis were measured as follows. Following treatment, cells were washed with PBS + 1%BSA and stained first with LIVE/DEAD™ Fixable Near‐IR Stain (Thermo Fisher Scientific, Waltham, MA, USA) 1 : 1000 in 100 µL for 30 min. at 4 °C. After washing cells in Annexin V binding buffer (cat. 556454, ABB; BD Pharmingen™, Franklin Lakes, NJ, USA) + 1% BSA, cells were stained with 5 µL PE‐conjugated Annexin V (cat. 556422; BD Pharmingen™) in a total of 100 µL for 15 min at room temperature. Cells were subsequently washed and fixated in ABB + 1% formaldehyde. Lastly, cells were washed and resuspended in ABB + 1% BSA. Apoptotic levels were quantified on a NovoCyte Flow Cytometer immediately after preparation. Quantification of surface CD20 levels was performed as follows. Cells were washed with PBS + 1% BSA and stained first with either LIVE/DEAD™ Fixable Near‐IR or LIVE/DEAD™ Fixable Violet Stain (Thermo Fisher Scientific) 1 : 1000 in 100 µL for 30 min at 4 °C. After washing the cells, they were stained with APC‐conjugated anti‐CD20 (cat. 55976; BD Pharmingen™), 15 µL in 100 µL for 30 min at 4 °C. Cells were subsequently washed and fixated in PBS + 1% formaldehyde. Lastly, cells were washed and resuspended in ABB + 1% BSA. Fluorescence was quantified on a NovoCyte Flow Cytometer (ACEA Biosciences, San Diego, CA, USA) or a LSRFortessa analyzer (BD Biosciences, Franklin Lakes, NJ, USA). For all stains, fluorescence minus one stains were also included.

### 2.9 Quantification of mRNA levels by RT‐qPCR

Cells were washed in PBS, and total RNA was isolated using chloroform and TRIzol® reagent (Thermo Fisher Scientific). For each sample, 5–10 × 10^6^ cells were harvested and lysed in 1 mL TRIzol; following the first chloroform separation (100 µL), a second separation with 300 µL of chloroform was performed. Total RNA was precipitated with isopropanol overnight at −80 °C, and the resulting pellet was washed in 70% ethanol and resuspended in 50–100 µL. All samples were at all times stored at −80 °C. Total RNA was treated with DNAse I (Thermo Fisher Scientific). First‐strand cDNA synthesis was performed using Maxima First Strand cDNA Synthesis for qPCR (Thermo Fisher Scientific) according to the manufacturer's protocol. Maxima Probe qPCR Master Mix (2×; Thermo Fisher Scientific) was used for reactions of 15 µL total. TaqMan® Assay primer‐probe set for MS4A1 (Hs0544819_m1) was used to detect MS4A1 mRNA levels. RPLP0 primers and probe sequences are available in Appendix S2. Reported MS4A1 *C*_t_ values are all relative to RPLP0, and the displayed values have been normalized to naïve cells.

### 2.10 Indel detection

The ‘indel rate’ for a cell population indicates the percentage of targeted alleles in the population carrying an insertion or a deletion potentially leading to gene KO. Indel rates as a measure of CRISPR‐directed KO efficiency were quantified by TIDE analysis. Primer and sgRNA sequences used for TIDE analysis are available in Appendix S2.

### 2.11 Western blot

Cells were counted, and 1 × 10^6^ cells were pelleted and washed twice in PBS. Cells were lysed in Pierce RIPA buffer (Thermo Fisher Scientific) with Roche Complete ULTRA Tablets protease inhibitor ×1 and 10 mm Naf. A total of 50 µL lysis buffer was used to 1 × 10^6^ cells, following incubation of the samples for 15 min on ice. Samples were then sonicated for 6 min corresponding to six cycles of 30 s on and 30 s off. After sonication, cell debris was pelleted by centrifugation at 14 000 ***g*** for 15 min. XT sample buffer 4X and DTT (Bio‐Rad, Hercules, CA, USA) were added, and samples were then incubated at 100 °C for 5 min. Equal volumes of each sample were loaded. Samples were separated on Criterion TGX Precast 18 well gels 10% (Bio‐Rad) in MOPS SDS Running Buffer (Thermo Fisher Scientific). The Precision Plus Protein™ All Blue Prestained Protein Standards (Bio‐Rad) ladder was loaded for size determination. Protein was transferred to Trans‐Blot® Turbo™ Mini PVDF membranes and blocked in washing buffer (TBS with 0.05% Tween‐20) with 5% skimmed milk. Primary antibodies were incubated overnight at 4 °C. BLNK was detected using BLNK(2B11) mouse monoclonal antibody (Santa Cruz Biotechnology, Dallas, TX USA) diluted 1 : 1000, BTK was detected using BTK (D3H5) rabbit monoclonal antibody (Cell Signaling and Technology) diluted 1 : 1000, and Vinculin was detected using Vinculin (V9131) mouse monoclonal antibody (Sigma‐Aldrich, St. Louis, MO, USA) diluted 1 : 10 000. After washing on the following day, membranes were incubated with secondary antibody HRP‐conjugated anti‐rabbit (P0448) or HRP‐conjugated anti‐mouse (P0447; Agilent Technologies). Membranes were visualized using Clarity Western ECL Substrate (Bio‐Rad). Uncropped western blot pictures are included in Figs S1–S11.

### 2.12 Figures and layout

Plots and graphs were created using either graphpad prism (GraphPad Software, San Diego, CA, USA) 8.0.2 or with R 3.5.1 (packages: ggplot2, gplots) layout, and final setup was done using Adobe Illustrator CC 2017 (Adobe, San Jose, CA, USA).

### 2.13 Statistical analysis

Statistical evaluation of library screen data was performed by MAGeCK as previously described [40]. Calculations from GSEA were performed by Enrichr as previously described [38, 39]. Experimental data were analyzed using graphpad prism 8.0.2. To determine statistical values in each experiment, Dunnett's multiple comparison test was performed. In each case, the multiple comparison test was carried out using the designated control sample from each experiment as control. The *P*‐values reported by Dunnett's multiple comparison test are multiplicity‐adjusted *P*‐values and not exact *P*‐values.

## 3 Results

### 3.1 Resistance of OCI‐Ly‐7 B cells harboring biallelic *MS4A1* knockout mutations in the presence and absence of active human serum

**Fig. 1:**
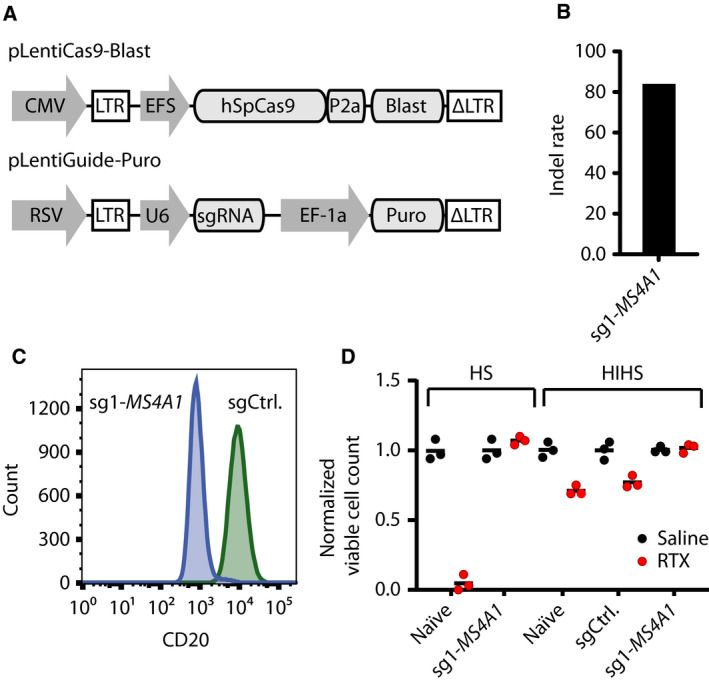
Absolute RTX resistance in OCI‐Ly‐7/*MS4A1* KO cells. (A) Schematics of vectors used to generate OCI‐Ly‐7‐Cas9 cells with stable SpCas9 expression and to deliver sgRNAs. (B) Indel rates in the OCI‐Ly‐7‐Cas9 9 days after delivery of a sgRNA targeting *MS4A1*. (C) Flow cytometric determination of CD20 protein levels in OCI‐Ly‐7‐Cas9 9 days after *MS4A1* sgRNA delivery. (D) RTX drug assay with either active or HIHS. Cells treated with 20% HIHS were grown in 50 µg·mL^−1^ RTX, whereas cells treated with 20% active HS were exposed to a RTX concentration of 10 µg·mL^−1^. Living cells were enumerated by trypan blue exclusion following 48 h of exposure. Black dots represent saline‐treated populations, whereas red dots display RTX‐treated populations. For each population of cells, living cells following treatment were normalized by dividing the number of cells with the mean of living cells counted in the saline‐treated cell population. For each population, treatment (saline or RTX) was carried out in triplicates; mean is shown.

To identify genes modulating the response of B cells to RTX, we set out to perform an unbiased genome‐wide CRISPR screen in OCI‐Ly‐7, a GCB‐subtype DLBCL cell line, using the Gecko v2 lentiviral library [30] consisting of 123 411 unique sgRNAs. It was initially validated that lentiviral vectors facilitate effective transfer of the CRISPR system to GCB‐subtype DLBCL cell lines (Fig. S1) and that endogenous CD20 expression was unaffected by this transfer method (Fig. S2). We then generated a OCI‐Ly‐7‐Cas9 clone stably expressing Streptococcus pyogenes Cas9 (SpCas9; Fig. 1A) and confirmed SpCas9 activity by lentiviral delivery of sgRNA targeting the *MS4A1* gene encoding CD20, leading to an indel rate of 85% (Fig. 1B) and strongly reduced CD20 expression (Fig. 1C). In the presence of HS providing human complement, OCI‐Ly‐7‐Cas9 cells carrying *MS4A1* KO mutations (OCI‐Ly‐7/*MS4A1*‐KO) were resistant to RTX, whereas naïve cells were drastically depleted primarily due to CDC (Fig. 1D). Also, naïve OCI‐Ly‐7‐Cas9 cells or cells stably expressing control sgRNA were sensitive to the direct effect of RTX in the presence of heat‐inactivated HS (HIHS), although the effect was less dramatic compared to conditions supporting CDC (Fig. 1D). Notably, OCI‐Ly‐7/*MS4A1*‐KO cells cultured in HIHS were resistant to RTX treatment. Collectively, these studies showed potent CRISPR‐directed KO in OCI‐Ly‐7‐Cas9 cells and confirmed the emergence of resistance to RTX upon cessation of CD20 expression.

### 3.2 Identification of rituximab response genes by genome‐wide CRISPR knockout screen in OCI‐Ly‐7 cells

**Fig. 2:**
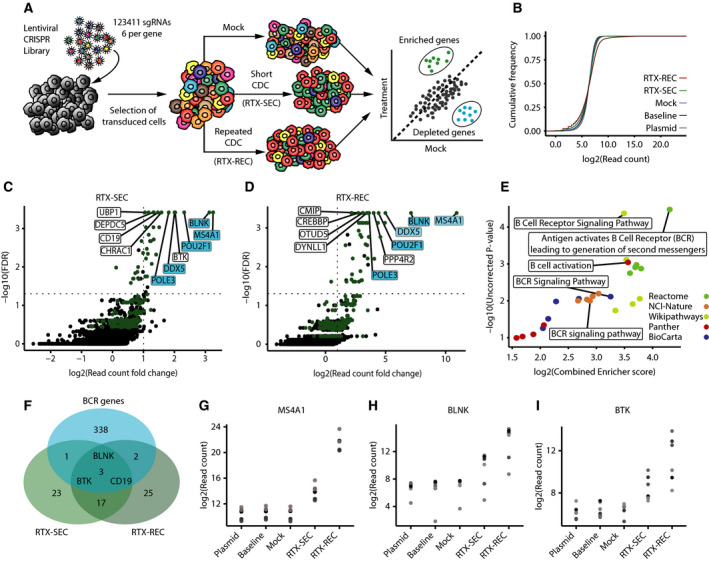
Genome‐wide CRISPR KO screening for identification of RTX response genes in OCI‐Ly‐7. (A) Schematic representation of screening strategy and concept. (B) Cumulative distribution of log_2_‐normalized sgRNA read counts for each sample. Skewing illustrates a higher percentage of sgRNAs with a low read count. (C, D) Results from MAGeCK analysis of low and high RTX conditions compared with mock treatment. The FDR (−log_10_) is plotted against the log_2_ fold change in read counts, relative to mock, across all sgRNAs targeting each gene. The 263 genes common for both RTX conditions (top 1000) are colored green, the top 10 genes for both RTX conditions are labeled, and the five genes common within the top 10 are highlighted in blue. (E) GSEA performed *via* Enricher, showing the top five pathways from five different databases. For each ranked pathway, the uncorrected *P*‐value (−log_10_) is plotted against the combined enricher score (log_2_). (F) Comparison of FDR cutoff genes from both RTX samples with a BCR gene set compiled from the various pathway databases. (G–I) *MS4A1*, *BLNK*, and *BTK* sgRNA read counts shown for the five samples including library plasmid preparation, baseline cells, mock‐treated cell population, and cells treated with low and high concentrations of RTX (RTX‐SEC and RTX‐REC). *Y*‐axis shows log_2_‐normalized read counts per sgRNA.

To screen the genome of OCI‐Ly‐7‐Cas9, cells were transduced with the lentiviral library (with a transductional titer of 4.7 × 10^7^ IU/mL; Fig. S3A) at a MOI of 0.5 (schematic overview in Fig. 2A). Whereas part of the cells were harvested as baseline control, the remaining cells were split in three populations that were subjected to three different treatment schemes: (i) mock, (ii) RTX‐SEC (followed by non‐CDC conditions for a total of 14 days), and (iii) RTX‐REC (maintained for 21 days). The rationale was to expose cells in the RTX‐SEC group shortly to CDC conditions followed by non‐CDC conditions, whereas cells in the RTX‐high group were repeatedly subjected to conditions supporting CDC (Fig. 1D). The overall distribution of sgRNA reads was determined by targeted NGS. Plotting the cumulative frequency of log_2_‐normalized read counts from all samples showed a distortion of the read distribution in the RTX‐REC group, whereas profiles for the remaining samples, including cells surviving the RTX‐SEC condition, were only slightly skewed relative to the profile of reads derived from sequencing of the plasmid library (Fig. 2B, Fig. S3B). Still, all samples contained reads from sgRNAs targeting at least 18 902 protein‐coding genes, corresponding to 99.2% of the original library. Using MAGeCK [40], sgRNA prevalence in each of the cell populations exposed to RTX was compared individually to mock, resulting in a ranking of each gene. By plotting FDR against the average sgRNA log_2_ fold change in read counts (Fig. 2C,D), highly ranked genes were separated from the bulk. With a FDR cutoff of 5%, sgRNAs targeting 44 genes were positively enriched in RTX‐SEC (Fig. 2C), whereas 47 genes, for which sgRNAs were enriched, emerged in RTX‐REC (Fig. 2D). A total of 20 genes scored below an FDR of 5% in both the RTX‐SEC and RTX‐REC groups, whereas a total of five genes (shown in light blue in Fig. 2C,D) were found among the top 10 enriched genes (indicated in Fig. 2C,D) for both conditions (exhaustive list of genes available in Appendix S1).

As expected from the critical role of CD20, sgRNAs targeting *MS4A1* were most prevalent in both RTX screens, verifying that KO mutations causing resistance to RTX were robustly pulled out from the RTX‐treated cell population. Notably, at RTX‐REC conditions, the raw *MS4A1* sgRNA read counts exceeded several millions (corresponding to 41% of all reads in the sample). Compared to RTX‐SEC conditions, the *MS4A1*‐targeting sgRNA sequences showed a 203‐fold higher enrichment in the RTX‐REC sample. Also, within the RTX‐REC group, *MS4A1*‐targeting sgRNAs showed a 13.93‐fold higher enrichment than sgRNAs targeting the second most enriched gene (*BLNK*), whereas the two genes were similarly enriched under RTX‐SEC conditions (1.08‐fold). In addition, we identified a strong enrichment of sgRNAs targeting *CREBBP* (a positive ranking of 11 and 9 at RTX‐SEC and RTX‐REC conditions, respectively). The *CREBBP* gene was recently reported as a regulator of CD20 expression [41] and claimed to be involved in lymphomagenesis [42]. Furthermore, our dataset showed enrichment of sgRNAs targeting *SPI1* (positive ranking of 41 and 21 at RTX‐SEC and RTX‐REC conditions, respectively) as well as depletion of sgRNAs targeting *FOXO1* (a negative ranking of 166 and 154 at RTX‐SEC and RTX‐REC conditions, respectively). These findings are in alignment with the notion that CD20 is repressed by binding of FOXO1 to the *MS4A1* promoter, as proposed by Scialdone and coworkers [41] and lend support to earlier findings showing that lower levels of *SPI1* expression are linked to decreased *MS4A1* transcription [43, 44], whereas reduced levels of FOXO1 are associated with increased *MS4A1* transcription [45]. Together, these observations not only emphasized the paramount impact of CD20 loss on the escape from RTX‐induced CDC, but also demonstrated the validity of the screen itself. Overall, we note that no other gene within the B‐cell genome has the substantial effect of CD20‐encoding *MS4A1* on RTX‐induced CDC.

Among the remaining genes, for which sgRNAs were enriched in the RTX‐resistant cell populations, we noted an overlap of 263 genes that were identified among the top 1000 most enriched genes from each of the two RTX conditions (Fig. 2C,D). We reasoned that these 263 genes were not directly related to resistance to CDC but rather reflected a group of genes affecting the direct response to RTX under complement‐depleted conditions. We explored this group of genes by GSEA using Enrichr [38, 39] and found that the 263 genes mapped mainly to pathways related to BCR signaling (Fig. 2E), suggesting that RTX mechanisms of action involve or interfere with BCR signaling. When genes with a FDR cutoff of 5% were cross‐referenced with a list of BCR‐related genes, we identified three genes, *BLNK*, *BTK*, and *CD19* encoding BLNK, BTK, and the B‐cell surface antigen CD19, respectively, as top‐ranking BCR signaling genes identified at both RTX‐SEC and RTX‐REC conditions (Fig. 2F). Lastly, we cross‐referenced our candidate genes with their scoring in mock sample (MAGeCK analysis of mock compared to baseline) and found that *CD19*‐targeting sgRNAs were depleted in the mock sample, implying that loss of CD19 had a negative impact on growth (scoring of mock sample genes available in Appendix S1). Hence, for *MS4A1* (Fig. 2G) as well as for *BLNK* and *BTK* (Fig. 2H,I), the library screen showed a robust enrichment of gene‐specific sgRNA sequencing reads in RTX‐resistant cells and no depletion within the mock sample. Notably, identification of *BLNK* (a positive ranking of 2 at both RTX‐SEC and RTX‐REC conditions) and *BTK* (a positive ranking of 5 and 19 at RTX‐SEC and RTX‐REC conditions, respectively) points to an important role of BCR signaling in relation to sensitivity to RTX under non‐CDC conditions.

### 3.3 Increased resistance to rituximab in B cells with knockout of *BLNK* or *BTK* genes

**Fig. 3:**
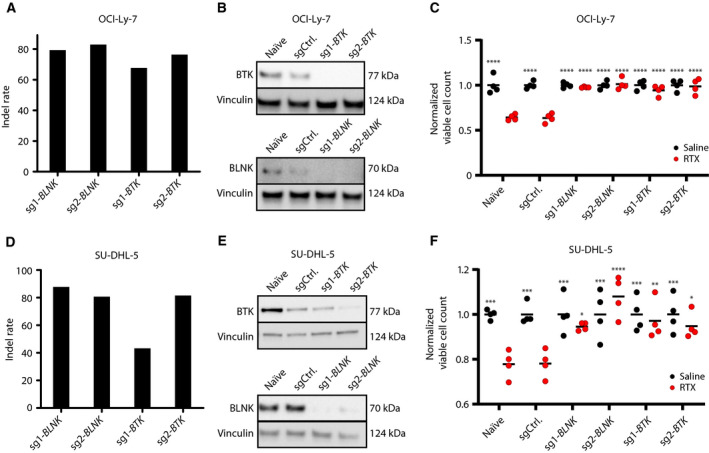
*BLNK* and *BTK* gene KO confers resistance to RTX in OCI‐Ly‐7. (A–C) and SU‐DHL‐5 (D–F) GCB subclass cells. (A, D) Assessment of CRISPR KO on the genomic level by TIDE. Following two weeks of puromycin, gDNA was harvested and sequence traces from sgRNA‐treated populations were compared by TIDE with sequences from cells treated with control sgRNA, allowing frequency of indels to be quantified. (B, E) Verification of *BLNK* and *BTK* KO assessed at the protein level by western blot. (C, F) RTX drug assay under non‐CDC conditions. Cells treated with 50 µg·mL^−1^ RTX in 20% HIHS were enumerated by trypan blue exclusion following 72 h of exposure. Black dots represent saline‐treated populations, whereas red dots display RTX‐treated populations. For each population of cells, living cells following treatment were normalized by dividing the number of cells with the mean of living cells counted in the saline‐treated cell population. For each population, treatment (saline or RTX) was carried out in triplicates; mean is shown. Dunnett's multiple comparison test was performed with RTX‐treated sgCtrl population (*< 0.05, **< 0.005, ***< 0.0005, ****< 0.0001).

Due to the tightly connected roles of BLNK and BTK during BCR signaling, we focused on confirming the correlation of the response to RTX with the status of the *BLNK* and *BTK* genes. We introduced KO mutations in the original OCI‐Ly‐7 cell line with a series of sgRNAs, two sgRNAs for both *BLNK* and *BTK*, resulting in cell populations with indel rates ranging from 60% to 80% (Fig. 3A) and shutdown of BLNK and BTK protein synthesis (Fig. 3B). Using growth conditions by which OCI‐Ly‐7 cells carrying a control sgRNA were exposed to RTX at a concentration of 50 µg·mL^−1^ for up to 72 h, we observed the most pronounced impact on cell viability after 72 h (Fig. S4). Using these growth conditions in the presence of HIHS, loss of BLNK or BTK protein resulted in markedly increased tolerance to RTX relative to naïve cells or cells expressing the control sgRNA (Fig. 3C). In parallel, using all four sgRNAs, we also introduced KO mutations in SU‐DHL‐5 cells, resulting in indel rates ranging from 40% to 88% (Fig. 3D) and suppressed protein levels (Fig. 3E). As in OCI‐Ly‐7 cells, KO of *BLNK* and *BTK* in SU‐DHL‐5 cells resulted in increased tolerance to RTX, as opposed to naïve cells and cells expressing a control sgRNA, which were both sensitive to the treatment (Fig. 3F).

To assess the impact of RTX in the context of ABC‐subtype cells lacking expression of *BLNK* and *BTK*, we aimed at introducing *MS4A1*, *BLNK*, and *BTK* KO mutations in RIVA cells, an ABC‐subtype cell line. However, whereas KO of *MS4A1* was successful, resulting in an indel rate of 81% (Fig. S5A), introduction of indels in *BLNK* and *BTK* severely reduced the proliferative potential of RIVA cells. Although a smaller fraction of cells eventually became resistant to the puromycin selection pressure, indel rates in these cells were very low, indicating that loss of *BLNK* or *BTK* in RIVA cells was incompatible with *in vitro* growth (Fig. S5A).

### 3.4 Relationship between surface‐expressed CD20 levels and rituximab‐induced apoptosis

**Fig. 4:**
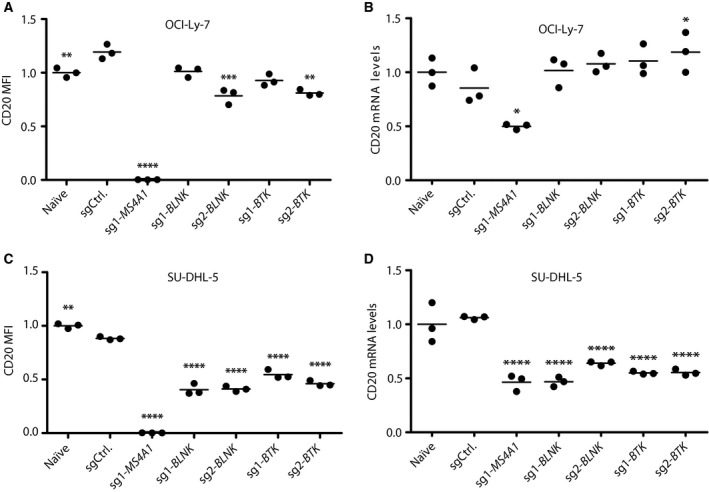
Cell type‐specific impact on CD20 expression levels in GCB‐subtype cells OCI‐Ly‐7. (A, B) and SU‐DHL‐5 (C, D) cells with KO of *BLNK* and *BTK*. (A, C) CD20 expression levels assessed by flow cytometry. Three separate samples from each population were prepared for flow. Illustrated ratios represent median fluorescent intensity of living cells relative to naïve expression level; shown is mean. (B, D) CD20 mRNA levels determined by qPCR. Three samples from each population were harvested, and for each, PCR was performed in duplicates for each sample. CD20 relative levels among samples were normalized to levels in naïve cells; shown is mean. *C*_t_ values were analyzed based on the standard curve method, and resulting relative values were used for Dunnett's multiple comparison test against the sgCtrl population (*< 0.05, **< 0.005, ***< 0.0005, ****< 0.0001).

As functions of BLNK and BTK could potentially affect CD20 cell surface expression, we assessed the CD20 levels in RTX‐resistant OCI‐Ly‐7/*BLNK*‐KO and OCI‐Ly‐7/*BTK*‐KO populations. CD20 levels in these cell populations were slightly reduced relative to sgCtrl‐expressing cells, but did not deviate from naïve cells (Fig. 4A). Also, *MS4A1* mRNA levels in OCI‐Ly‐7/*BLNK*‐KO and OCI‐Ly‐7/*BTK*‐KO cells were unaffected (Fig. 4B), whereas *MS4A1* mRNA levels in OCI‐Ly‐7/*MS4A1‐* KO cells were reduced to 50% of the level in sgCtrl‐expressing cells, most likely due to the introduction of indels. In contrast, CD20 levels at the surface of SU‐DHL‐5/*BLNK*‐KO and SU‐DHL‐5/*BTK*‐KO cells were markedly reduced (40–54% of the level in sgCtrl‐treated cells; Fig. 4C). As the *MS4A1* mRNA levels were reduced correspondingly across all KO populations to levels mimicking the level in SU‐DHL‐5/*MS4A1* KO cells (Fig. 4D), it was noted that KO of *BLNK* as well as of *BTK* in SU‐DHL‐5 cells resulted in the reduction of CD20 presentation on the cell surface, most likely through mechanisms involving transcriptional and/or post‐transcriptional regulation.

**Fig. 5:**
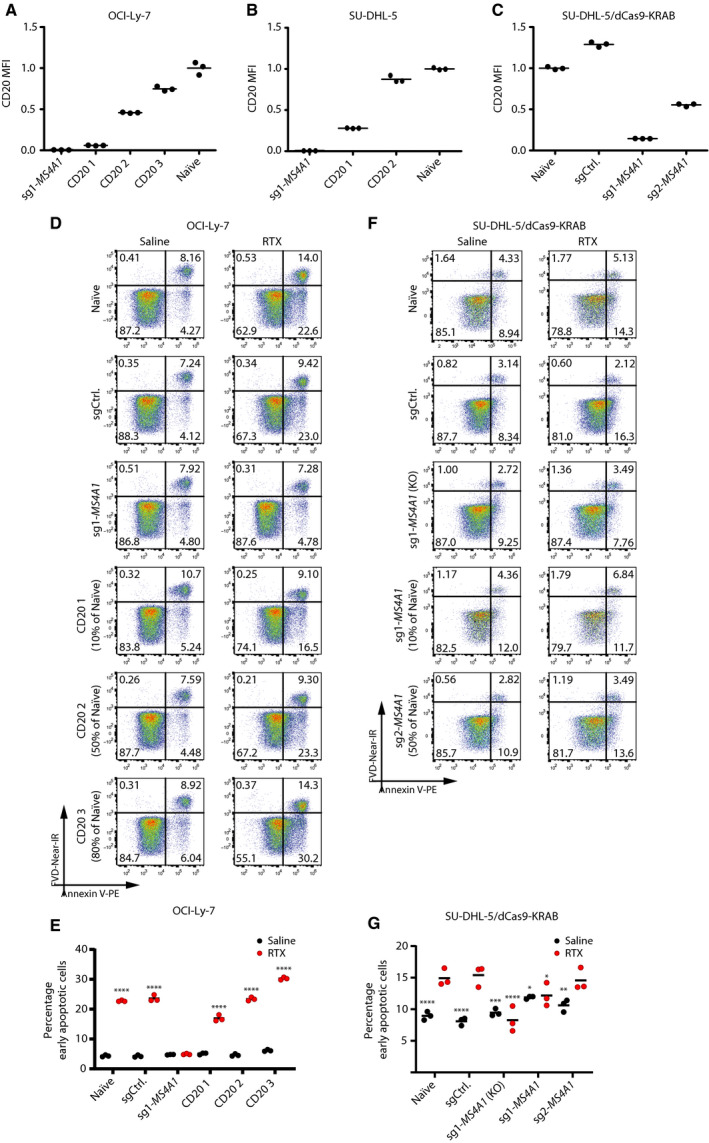
RTX response in relation to CD20 surface levels. (A, B) Surface expression levels of CD20 in OCI‐Ly‐7 (A) and SU‐DHL‐5 (B) following multiple lentiviral transductions with LV/PGK‐CD20; three separate samples from each population were prepared for flow. Illustrated ratios represent median fluorescent intensity relative to naïve; shown is mean. (C) CD20 surface expression in SU‐DHL‐5/KRAB cells transduced with either control sgRNA or sgRNAs targeting *MS4A1*. CD20 expression was quantified by flow cytometry; from each population, triplicates were prepared for flow. Illustrated ratios represent median fluorescent intensity relative to the untreated population; shown is mean. (D) Representative plots from flow cytometric assessment of apoptosis in OCI‐Ly‐7 CD20‐reconstituted populations treated with 50 µg·mL^−1^ RTX and 20% HIHS for 24 h. (E) Summarized percentage of early apoptotic cells of OCI‐Ly‐7 populations with reconstituted CD20 levels. Black dots represent the saline‐treated populations, whereas red dots display RTX‐treated populations. Dunnett's multiple comparison test was performed with RTX‐treated *MS4A1‐*sg1 population. (F) Representative plots from flow cytometric assessment of apoptosis in SU‐DHL‐5/KRAB *MS4A1* inhibition populations following 48 h of treatment with 50 µg·mL^−1^ RTX and 20% HIHS. (G) Summarized percentage of early apoptotic cells of SU‐DHL‐5/KRAB populations with suppressed expression of the *MS4A1* gene. Black dots represent saline‐treated populations, whereas red dots display RTX‐treated populations. For each population, treatments (saline or RTX) were carried out in triplicates; mean is shown. Dunnett's multiple comparison test was performed with RTX‐treated sgCtrl population (*< 0.05, **< 0.005, ***< 0.0005, ****< 0.0001).

To explore whether variations in cell surface expression of CD20 affected RTX‐induced apoptosis in BLNK‐ and BTK‐deficient B cells, we set out to reconstitute CD20 expression levels in *MS4A1*KO cells by transduction of the cells with a lentiviral vector encoding a sgMS4A1‐resistant CD20 variant (Fig. S6). OCI‐Ly‐7/*MS4A1*‐KO and SU‐DHL‐5/*MS4A1*‐KO cells were therefore transduced successively (three and two transductions, respectively), leading to gradually increased CD20 levels spanning from 10% to 80% of the normal level (Fig. 5A,B). To establish a population of SU‐DHL‐5 cells with the same level of CD20 expression as in SU‐DHL‐5/*BLNK*‐KO and SU‐DHL‐5/*BTK*‐KO (40–50% of normal), we suppressed the transcriptional activity of the *MS4A1* locus using an RNA‐guided SpCas9‐KRAB fusion variant (Fig. S6), resulting in a CD20 expression level (50% of normal; Fig. 5C) that mimicked the level in SU‐DHL‐5/*BLNK*‐KO and SU‐DHL‐5/*BTK*‐KO cells (Fig. 4C).

We investigated the impact of RTX on B cells by quantifying early and late apoptotic cells over time (Fig. S7) and noticed the largest impact of RTX on the early apoptotic fraction of cells quantified after 24 h. Hence, the percentage of early apoptotic naïve OCI‐Ly‐7 cells after exposure to RTX for 24 h was increased from 9.5 ± 1.1 to 29.2 ± 1.6% (Fig. S8). Similar induction was seen in cells expressing the control sgRNA, whereas induction of apoptosis was blocked as expected in OCI‐Ly‐7/*MS4A1*‐KO cells due to the absence of CD20. In cells with 10% of the normal CD20 expression, 16.5 ± 0.9% of the cells were early apoptotic, whereas 30.2 ± 0.4% of the cells with 80% of normal CD20 presentation reached early apoptosis (Fig. 5D). Upon treatment of naïve and control cells with RTX, the percentage of early apoptotic cells was increased, as expected, whereas OCI‐Ly‐7/*MS4A1*‐KO cells were unaffected by the treatment (Fig. 5D). Importantly, the basic level of apoptosis corresponding to the level in naïve cells was reached already in cells with CD20 expression levels reaching 50% of normal (Fig. 5E). In SU‐DHL‐5 cells, in which the CD20 level was suppressed to 50% of normal, RTX‐induced early apoptosis did not deviate from the level in control cells (Fig. 5F,G), whereas the capacity to induce apoptosis in cells expressing 10% of normal CD20 levels did not differ from the capacity in SU‐DHL‐5/*MS4A1*‐KO cells (Fig. 5F). In both OCI‐Ly‐7 and SU‐DHL‐5/dCas9‐KRAB cells, the same tendency was evident in the late apoptotic cells, but to a smaller extent (Fig. S9) as was expected from our initial quantification of late apoptotic cells appearing after treatment with RTX (Fig. S7). In summary, our data demonstrate that the direct effect of RTX under non‐CDC conditions correlates with CD20 levels. However, when we considered the response toward RTX in cells in which the CD20 expression level was artificially engineered to mimic the levels in cells with *BLNK* and *BTK* KO, the reduced levels of CD20 expression measured in cells carrying KO mutations of *BLNK* or *BTK* cells did not alone explain the altered response to RTX. We therefore conclude that sgRNAs targeting *BLNK* and *BTK* were not solely enriched in genome‐wide screens through mechanisms involving modulation of the CD20 expression levels.

### 3.5 Impairment of rituximab‐directed apoptosis in *BLNK* and *BTK* knockout populations

**Fig. 6:**
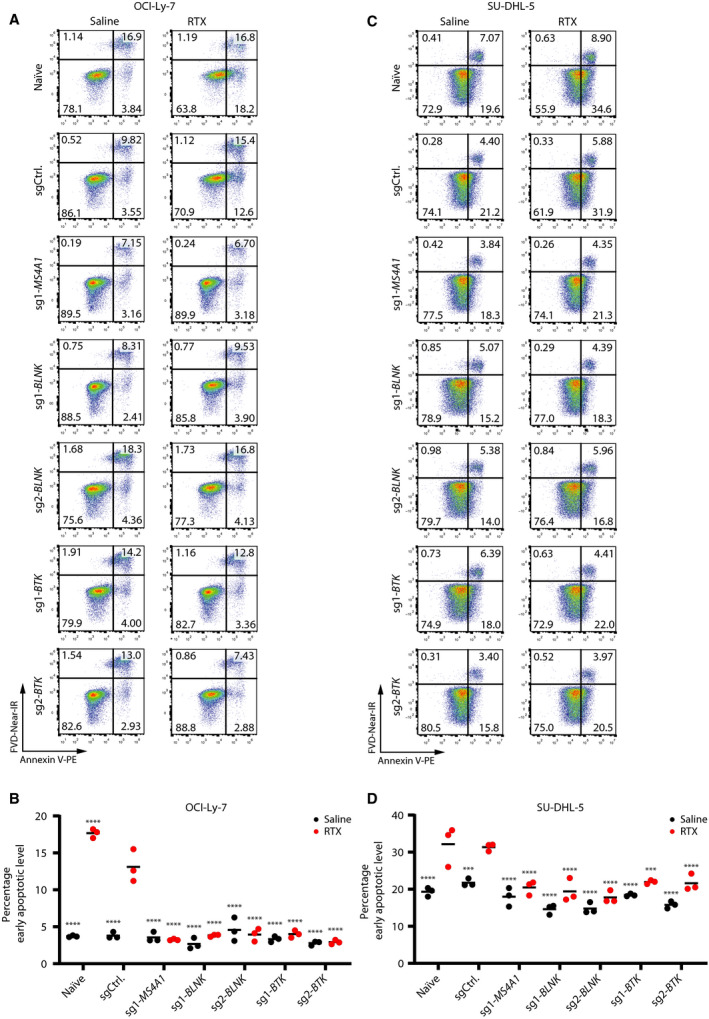
Absence of RTX‐induced apoptosis in *BLNK* and *BTK* KO OCI‐Ly‐7. (A, B) and SU‐DHL‐5 cells (C, D). (A, C) RTX apoptosis assay with non‐CDC conditions; representative samples are shown. Cells were treated with 50 µg·mL^−1^ RTX in 20% HIHS. Levels of apoptosis were determined by annexin V and live/dead staining. OCI‐Ly‐7 cells were exposed to RTX for 24 h and SU‐DHL‐5 cells for 48 h. (B, D) Summarized percentage of early apoptotic cells. Black dots represent saline‐treated populations, whereas red dots display RTX‐treated populations. For each population, treatments (saline or RTX) were carried out in triplicates; mean is shown. Dunnett's multiple comparison test was performed with RTX‐treated sgCtrl population (***< 0.0005, ****< 0.0001).

To further investigate the acquired resistance to RTX in OCI‐Ly‐7/*BLNK*‐KO and OCI‐Ly‐7/*BTK*‐KO cells, we determined levels of apoptosis after subjecting the cells to RTX under non‐CDC conditions. After exposure of OCI‐Ly‐7 cells to RTX for 24 h, we did not find indications of induced apoptosis, leading to early apoptotic cells neither in the two *BLNK* KO populations nor in the two *BTK* KO populations (Fig. 6A). Hence, the percentage of early apoptotic cells in OCI‐Ly‐7/*BLNK‐*KO (3.91 ± 0.1% for sg1‐BLNK) and *BTK* KO (4.01 ± 0.4% for sg1‐BTK) mimicked the level in OCI‐Ly‐7/*MS4A1*‐KO (3.24 ± 0.1%), which was substantially lower than the level reached in control cells (13.1 ± 1.8%; Fig. 6B). In OCI‐Ly‐7 cells, the same trend was evident in the fraction of late apoptotic cells after RTX treatment (Fig. S10A). Additionally, for OCI‐Ly‐7 we found the same effect on the apoptotic response after 48 and 72 h (Fig. S11). In SU‐DHL‐5 cells, the percentage of early apoptotic cells increased upon exposure to the drug for 48 h, although the basic level of the annexin V stain was higher in these cells (Fig. 6C). Notably, relative to sgRNA control cells, SU‐DHL‐5/*MS4A1*‐KO cells did not undergo induced apoptosis upon exposure to RTX (Fig. 6D). Similarly, treatment of SU‐DHL‐5/*BLNK*‐KO and SU‐DHL‐5/*BTK*‐KO cells with RTX did not increase the early apoptotic levels beyond the levels observed in the absence of RTX (Fig. 6C,D), suggesting that the lack of BLNK and BTK rendered the cells unable to undergo programmed cell death induced by RTX. A similar lack of response was evident in the fraction of late apoptotic cells after RTX treatment (Fig. S10B). Altogether, our findings demonstrate that BCR signaling through BTK and BLNK is essential for RTX‐induced signaling and subsequent apoptosis in GCB‐subtype DLBCL cells.

## 4 Discussion

B‐cell proliferation and differentiation is driven by multibranched intracellular signaling cascades propagating through BCR antigen recognition. As a key regulator of cell growth, BCR signaling is vulnerable for irregularities driving outgrowth of malignant B cells, as evident in DLBCL [46, 47]. In ABC‐subtype DLBCL, malignancy is driven by antigen‐induced BCR activation [48], whereas signaling through BCR in GCB‐subtype DLBCL is suggested to occur independently of antigen recognition through ‘tonic’ mechanisms [49]. Despite the key function of BCR in triggering proliferative growth signals that may potentially be malignant, signaling through BCR may also be crucial in attempts to treat B‐cell cancer with R‐CHOP. Hence, it is now generally accepted that intracellular signals driven by the BCR are directly affected by the binding of RTX to CD20 with direct effects on cell growth and survival [18, 19, 50]. Here, we set out to screen the genome of OCI‐Ly‐7, a GCB‐subtype B‐cell line, for genes related to resistance toward RTX using a lentivirus‐based genome‐wide CRISPR/Cas9 screening approach. By applying different RTX selection modalities to a large heterologous population of cells, with each cell carrying CRISPR‐induced gene‐disruptive indels in a single gene, we identified genes with functions that support sensitivity to RTX. Under conditions where the library‐treated population of OCI‐Ly7 cells was exposed to RTX, we consistently found sgRNAs targeting the *MS4A1* gene as predominantly enriched, supporting that CD20 is essential for the cytotoxic impact of RTX. In addition to the expected identification of *MS4A1,* the rankings of *CREBBP*, *SPI1*, and *FOXO1* within our screening data support the notion that CREBBP and SPI1 are involved in promoting CD20 expression and FOXO1 repression by binding to the *MS4A1* promoter, a model proposed by Scialdone and coworkers [41]. Previously, *MS4A1* expression has been investigated by shRNA‐based screening of the genome [51] showing that *SPI1* was also in this study linked to reduced *MS4A1* expression. Overall, such observations support the validity of our approach and may potentially suggest that several of the remaining identified genes play roles in modulating *MS4A1* expression.

In OCI‐Ly‐7 and SU‐DHL‐5, both GCB cell lines, we successfully validated that KO of the *BLNK* and *BTK* genes resulted in induced resistance to RTX, leading to increased cell viability. Such resistance was more pronounced in OCI‐Ly‐7 as compared to SU‐DHL‐5, an observation possibly explained by a difference in the intrinsic sensitivity toward RTX under non‐CDC conditions. This is in line with previous studies reporting that OCI‐Ly‐7 cells are more sensitive toward RTX under non‐CDC conditions than SU‐DHL‐5 cells [52]. To assess the impact of lost *BLNK* and *BTK* gene function in the ABC context, we made attempts to disrupt these genes in RIVA, an ABC cell line. However, treatment of RIVA cells with sgRNAs targeting *BLNK* and *BTK* severely hindered proliferation of RIVA cells, and the surviving population of cells was enriched for cells without *BLNK* and *BTK* KO mutations. This supports previous findings showing that growth of ABC subclass cells depends on BCR signaling feeding into a constant hyperactivation of NF‐ΚB signaling [48]. Notably, this also indicates that the unique involvement of *BLNK* and *BTK* in shaping the resistance to RTX, without being crucial for cell growth, could potentially apply exclusively to GCB‐subtype cells.

Due to the well‐established function of BLNK and BTK proteins as key players in BCR signaling pathways, we explored our screen data with focus on other core BCR genes, acting upstream of BLNK and BTK. Notably, neither *CD79A* nor *CD79B*, encoding proteins which both which act immediately downstream from the BCR [53], were among the top‐ranked genes in the screen (a positive ranking of 6953 and 18 187 of CD79A and CD79B, respectively, in the RTX‐SEC condition) or in the mock sample (a positive ranking of 15 273 and 18 843 of CD79A and CD79B, respectively). The low ranking of the *CD79A* and *CD79B* genes is in line with the low ranking of CD19 (a negative ranking of 829 in the mock sample) and could indicate that KO of these as well as other key BCR signaling genes directly affected cell growth or survival under normal conditions. These findings are in accordance with recent findings showing that GCB cell lines depend on ‘tonic’ BCR‐induced signaling through PI3K/AKT [49]. Importantly, such tonic BCR signaling in GCB‐subtype cells seems to occur independently of the BLNK and BTK proteins, whereas cessation of *BLNK* and *BTK* gene expression interferes with signaling pathways that are normally affiliated with RTX‐induced intracellular signals leading to constrained cell growth or apoptosis.

Considering the specificity of RTX for CD20, the impact of the *BLNK* and *BTK* genes on RTX resistance suggests that stimulation of CD20 feeds into BCR signaling, a claim which is also supported by recent publications linking RTX response in chronic lymphocytic leukemia to BCR signal‐proficient cell populations [54] and studies of RTX‐induced BCR signaling in follicular lymphoma cell lines [21]. As expected, we found that cessation of CD20 surface expression led to complete RTX tolerance both in relation to CDC and direct intracellular effects. CD20 is involved in calcium‐related signaling and plays an important role in T‐cell independent activation of B cells [55], which could explain its close relations to the BCR. Also, previous reports have shown reduced expression of BCR and decreased BCR signaling in CD20^‐/‐^ mice [56]. This connection is supported by association between expression of the *MS4A1* gene and expression of several BCR‐related genes in patient samples [57] and evidence of physical association with the BCR [58]. The direct association between CD20 and BCR has previously been linked to aggregation of membrane lipid rafts [59, 60], a theory supported by the recent resolution crystal structure of RTX bound to CD20, demonstrating CD20 super assemblies in the cell membrane [61]. In addition to a potential impact of CD20 on BCR signaling, the exposure of cells to antibodies directed at CD20 has previously been reported to elicit changes in BCR signaling. For instance, CD20 stimulation was shown to induce phosphorylation of PLCG2 (gene positively ranked 57 and 111 at RTX‐SEC and RTX‐REC conditions, respectively), a protein in direct interaction with BLNK and BTK as part of BCR signaling [62]. Furthermore, stimulation by RTX and obinutuzumab (a third‐generation anti‐CD20 antibody) was compared by global phosphoproteomics, revealing that phosphorylation of several proteins involved in BCR signaling was induced by both antibodies [50]. Of particular interest, a direct association of CD20 with the BCR and subsequent phosphorylation of BLNK following stimulation of CD20 has been linked to calcium signaling [17]. Calcium signaling following treatment with RTX has previously been connected to apoptosis, evident by the effects of inhibiting store‐operated calcium entry [16]. Also, studies have linked treatment with RTX to activation of BCR‐dependent induction of apoptosis. Specifically, apoptosis induced by CD20 stimulation was shown to be mediated through caspase‐3 [18, 19]. As expected, we observed an increase in apoptotic levels in unmodified B‐cell lines exposed to RTX. In contrast, *MS4A1* KO populations showed no induction of apoptosis. Notably, KO of *BLNK* and *BTK* resulted in the exact same phenotype rendering the GCB‐type B‐cell lines resistant to RTX‐induced apoptosis. Collectively, our data demonstrate the action of RTX through BCR signaling leads to apoptosis.

Despite shared pathways between CD20‐ and BCR‐directed intracellular signaling, CD20 remains the gateway to RTX‐mediated effects. As such, resistance toward RTX‐induced CDC has previously been investigated in relation to *MS4A1* expression level [52, 63, 64, 65]. Therefore, we assessed the *MS4A1* expression levels in OCI‐Ly‐7 and SU‐DHL‐5 cells following *BLNK* and *BTK* KO. Expression of CD20 was only vaguely reduced in OCI‐Ly‐7 cells upon *BLNK* and *BTK* KO, whereas lack of BLNK or BTK led to a marked reduction of CD20 levels in SU‐DHL‐5 cells. Our observations are in line with previous studies showing that interference with BCR signaling, by treatment with SRC family kinase inhibitors and BTK inhibitors, leads to reduced *MS4A1* expression [66, 67, 68, 69].

By generating a range of cell lines expressing CD20 at levels ranging from 5% to 80% of the level in naïve cells, we observed a correlation between the level of CD20 and the effect of RTX under non‐CDC conditions, as has previously been observed [21]. However, at CD20 expression levels comparable to those observed in *BLNK* KO and *BTK* KO cells, we observed no difference in apoptosis induction (Fig. 5E,G). Combined with the fact that OCI‐Ly‐7 cells show the least reduction in CD20 levels and yet acquire the most pronounced RTX resistance following loss of BLNK and BTK, altered CD20 levels due to the lack of BLNK and BTK did not alone explain the acquired resistance, suggesting that RTX‐induced apoptosis was mediated by intracellular signaling through BLNK and BTK.

## 5 Conclusions

In combination with previous studies on the effect of BTK inhibitors on RTX sensitivity [66, 67, 68, 69], our findings could potentially point to conflicting consequences related to the use of BTK or other BCR inhibitors to RTX‐containing regimes. One may ask whether inhibition of BTK is likely to increase B‐cell resistance to RTX? As illustrated by the differences among GCB and ABC subtypes in relation to dependency of BLNK and BTK, this potential contraindication may prove also to be subtype‐specific. Analysis of such differences between GCB and ABC subtypes could potentially give insight to explain why attempts to treat DLBCL patients, specifically based on GCB and ABC subtypes, with novel compounds have in large failed.

In conclusion, our data support that CD20‐dependent RTX‐induced signaling under non‐CDC conditions feeds into BCR signaling and reveal a GCB subclass‐specific gain of RTX resistance caused by both complete shutdown of RTX‐induced apoptosis pathways and reduced CD20 expression following loss of BLNK and BTK.

## Author contributions

EAT and JGM drafted and designed the study with assistance from KD and HD. EAT and ABR conducted the experiments. JH and YL performed next‐generation sequencing. EAT and MVA performed bioinformatics analyses. EAT analyzed the data. KD contributed to reagents/materials. EAT and JGM wrote the manuscript and prepared the figures. All authors have read and approved the final manuscript.

## Conflicts of interest

The authors declare no conflict of interest.

## Supporting information


**Fig. S1.** Efficient lentiviral transfer to malignant B‐cells at low MOI. (A) Schematic representation of vectors pCCL/PGK‐eGFP and pLentiCRISPRv2, the latter which was used to produce KO cell lines. (B) Transductional titers of LV/PGK‐eGFP and LV/CRISPRv2 in OCI‐Ly‐7 cells. Copy numbers following transduction were determined by qPCR; number of transducing units per milliliter is displayed. Three separate transductions were performed, and qPCR were performed in technical duplicates; shown is mean. (C) Median enhanced green fluorescent protein (eGFP) fluorescence in OCI‐Ly‐7 and SU‐DHL‐5 cells following transduction with different doses of LV/PGK‐eGFP. MOI estimates are based on qPCR. (D) Percentage of eGFP‐positive OCI‐Ly‐7 and SU‐DHL‐5 cells following transduction with different doses of LV/PGK‐eGFP. MOI estimates are based on qPCR; three separate samples from each population were prepared for flow. Mean is shown.
**Fig. S2.** Lentiviral gene delivery to B‐cell lines does not affect CD20 surface levels. CD20 surface expression in OCI‐Ly‐7 (A) and SU‐DHL‐5 (B) populations transduced with different lentiviral constructs. Three transductions per dose were performed. Illustrated ratio represent median CD20 fluorescent intensity relative to naïve population; shown is mean. (C) Gating strategy for flow cytometric assessment of CD20 expression levels.
**Fig. S3.** CRISPR library delivery and representation. (A) Transduction titer of lentiviral preparation used to deliver the Gecko v2 CRISPR library. Copy numbers following transduction were determined by qPCR; number of transducing units per milliliter is displayed. Three separate transductions were performed, and qPCR were performed in technical duplicates. Shown is mean. (B) Violin plots of sgRNA read counts across all samples; log_2_‐normalized read counts have been plotted.
**Fig. S4.** Response to rituximab dependent on exposure time. Rituximab drug assay with non‐CDC conditions in OCI‐Ly‐7 cells expressing control sgRNA. Cells were treated with 50 µg·mL^−1^ rituximab in 20% HIHS and counted using trypan‐blue exclusion following 24, 48 and 72 h of incubation. Black dots represent saline‐treated populations, whereas red dots display rituximab‐treated populations. For each population of cells, living cells following treatment was normalized by dividing the number of cells with the mean of living cells counted in the saline‐treated cell population. For each population, treatment (saline or rituximab) was carried out in triplicates; mean is shown.
**Fig. S5.** Failure to KO *BLNK* and *BTK* genes in ABC‐subclass cell line RIVA. (A) TIDE analysis of *MS4A1, BLNK* and *BTK* genes in RIVA cells from one of the attempts. Attempts were made to KO *BLNK* and *BTK* with two individual sgRNAs per gene along with successful KO of *MS4A1*.
**Fig. S6.** Modulation of CD20 levels by successive transductions and CRISPR‐based inhibition of *MS4A1* transcription. (A) Schematic representation of pCCL/PGK‐MS4A1 with details on silent mutations introduced to abolish the sgRNA target site and pLX‐331‐KRAB‐Cas9 (used to produce SU‐DHL‐5 cells with stable expression of KRAB‐fused hSpCas9). Percentage of OCI‐Ly‐7 CD20 positive cells (B) and CD20 median fluorescent intensity (C) following transduction with different doses of LV/PGK‐CD20. Percentage of SU‐DHL‐5 CD20 positive cells (D) and CD20 median fluorescent intensity (E) following transduction with different doses of LV/PGK‐CD20. Three separate samples from each population were prepared for flow. Illustrated ratios represent median fluorescent intensity relative to naïve cells, shown is mean and.
**Fig. S7.** Level of rituximab‐induced apoptosis depends on exposure time. Cells were treated with 50 µg·mL^−1^ rituximab in 20% HIHS for 24, 48, and 72 h. Apoptotic levels were determined by annexin V and live/dead staining. Early and late apoptotic levels in OCI‐Ly‐7 cells expressing control sgRNA (A, B) and in SU‐DHL‐5 cells expressing control sgRNA (C, D). Summarized percentage of apoptotic cells. Black dots represent saline‐treated populations, whereas red dots display rituximab‐treated populations. For each population, treatment (saline or rituximab) were carried out in triplicates; mean is shown. (E) Gating strategy for flow cytometric detection of apoptotic levels.
**Fig. S8.** Complete blockage of rituximab‐induced apoptosis in OCI‐Ly7 cells following *MS4A1* KO. (A) Representative plots of apoptosis assay in OCI‐Ly‐7 cells. Cells were treated with 50 µg·mL^−1^ rituximab in 20% HIHS for 24 h. Apoptotic levels were determined by annexin V and live/dead staining. (B) Summarized percentage of early apoptotic cells. Black dots represent saline‐treated populations, whereas red dots display rituximab‐treated populations. For each population, treatment (saline or rituximab) were carried out in triplicates; mean is shown.
**Fig. S9.** Percentage of late apoptotic cells at varying levels of CD20 cells following exposure to rituximab. (A) OCI‐Ly‐7 and (B) SU‐DHL‐5/dCas9‐KRAB. Black dots represent saline‐treated populations, whereas red dots display rituximab‐treated populations. For each population, treatment (saline or rituximab) were carried out in triplicates; mean is shown.
**Fig. S10.** Percentage of late apoptotic cells following 24 h of rituximab. (A) OCI‐Ly‐7 and (B) SU‐DHL‐5. Black dots represent saline‐treated populations, whereas red dots display rituximab‐treated populations. For each population, treatment (saline or rituximab) were carried out in triplicates; mean is shown.
**Fig. S11.** Apoptotic levels in OCI‐Ly‐7 cells following longer exposure to rituximab. OCI‐Ly‐7 cells were treated with 50 µg·mL^−1^ rituximab in 20% HIHS for 48 h (A, B) or 72 h (C, D). Apoptotic levels were determined by annexin V and live/dead staining. For each population, three treatments (saline or rituximab) were carried out. (A, C) summarized percentage of early apoptotic cells, (B, D) summarized percentage of late apoptotic cells. Black dots correspond to saline‐treated populations, whereas red dots display rituximab treated populations. For each population, treatment (saline or rituximab) were carried out in triplicates; mean is shown.

**Appendix S1.** CRISPR screen data. Analyzed data from genome‐wide CRISPR screen.

**Appendix S2.** BCR gene set and oligos. Gene set of 263 commonly enriched genes submitted for EnrichR analysis and sgRNA and TIDE primer sequences.

**Appendix S3.** Raw data for all figures. Excel spreadsheet with data used for calculations and plotting related to each figure.

## Data Availability

Raw data from all figures are included in Appendix S3. The sequencing data from our genome‐wide CRISPR screen is available at NCBI gene expression omnibus, under accession number GSE139385.
