# Supplementary material for: Identification of BLNK and BTK as mediators of rituximab‐induced programmed cell death by CRISPR screens in GCB‐subtype diffuse large B‐cell lymphoma
Source: Mol Oncol. 2020 Jul 16;14(9):1978–97. doi: 10.1002/1878-0261.12753 (PMC7463323; doi:10.1002/1878-0261.12753)

Supplemental figure S1

A

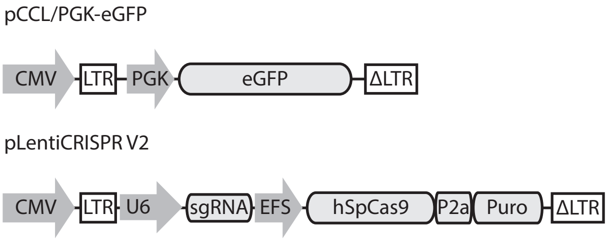

B

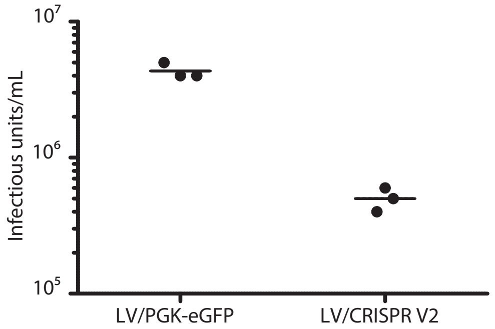

C

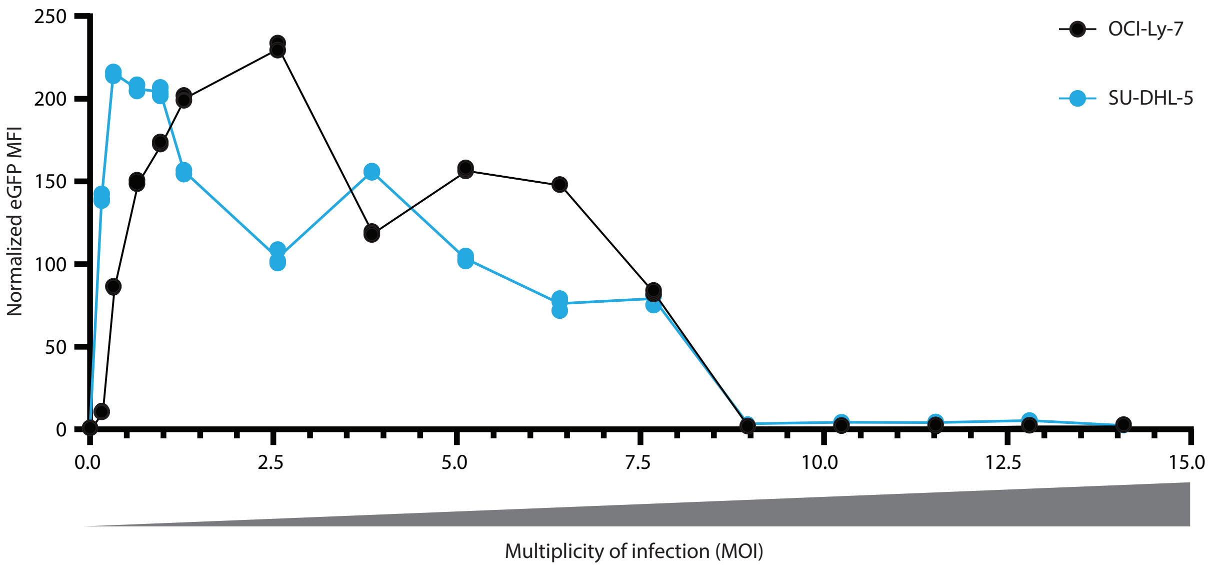

D

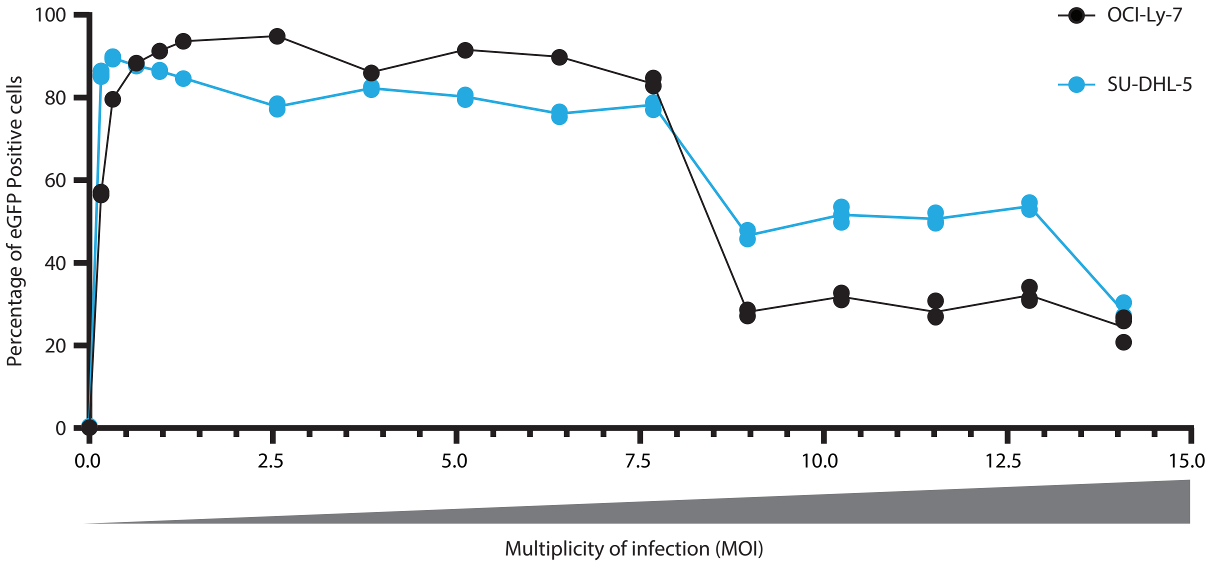

Supplemental figure S2

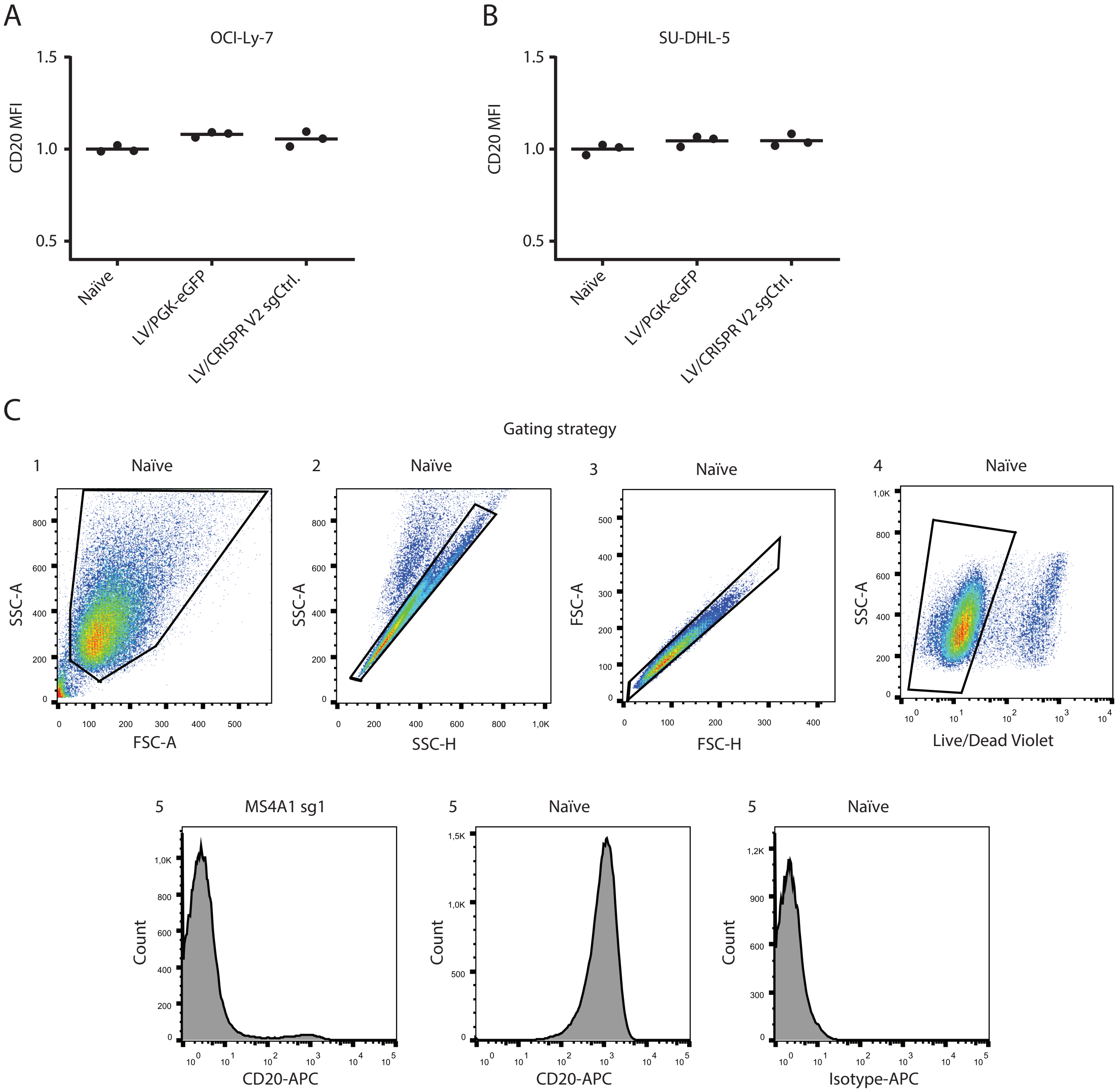

Supplemental figure S3

A

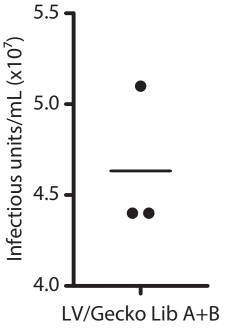

B

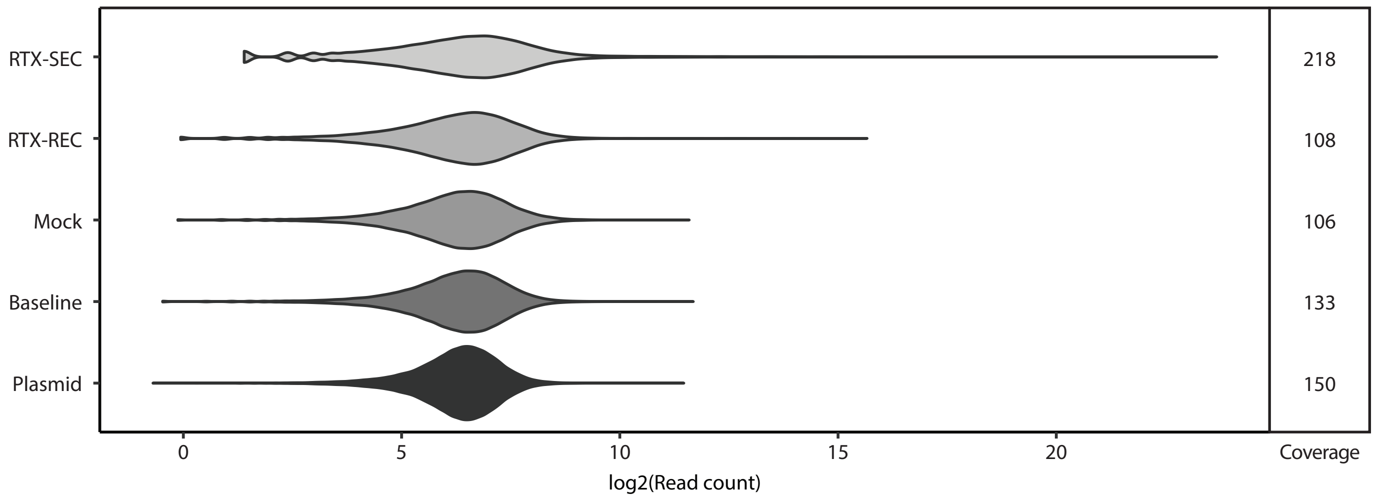

Supplemental figure S4

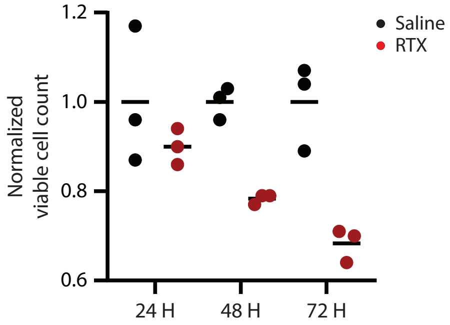

Supplemental figure S5

A

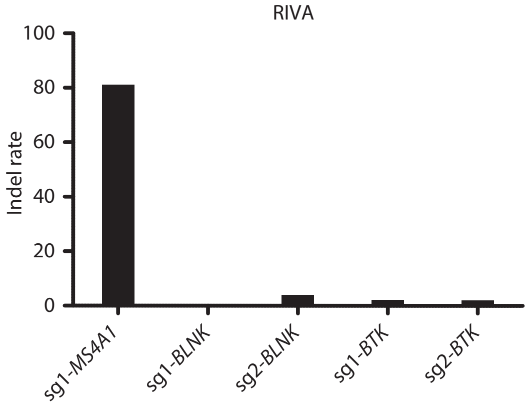

Supplemental figure S6

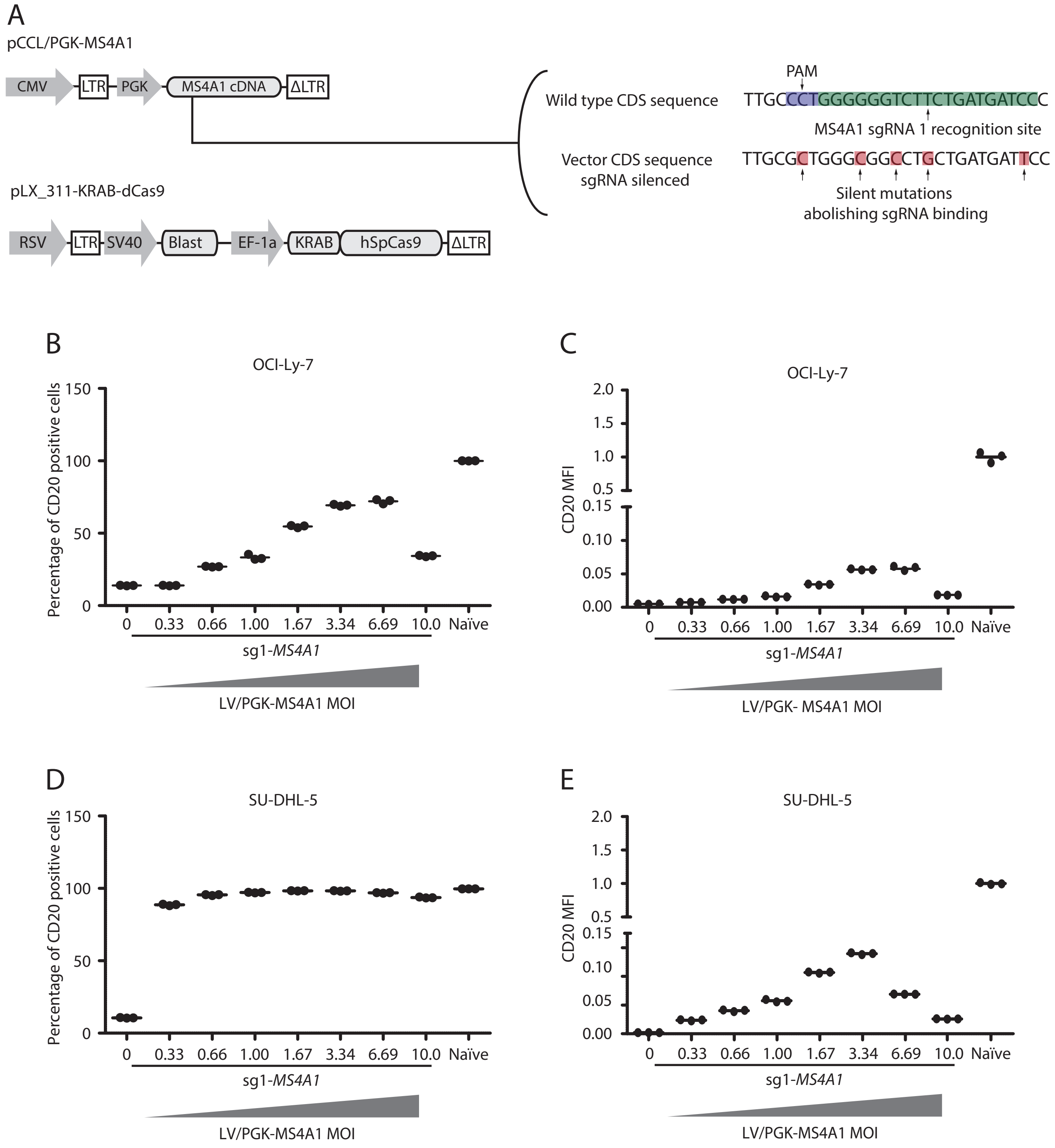

Supplemental figure S7

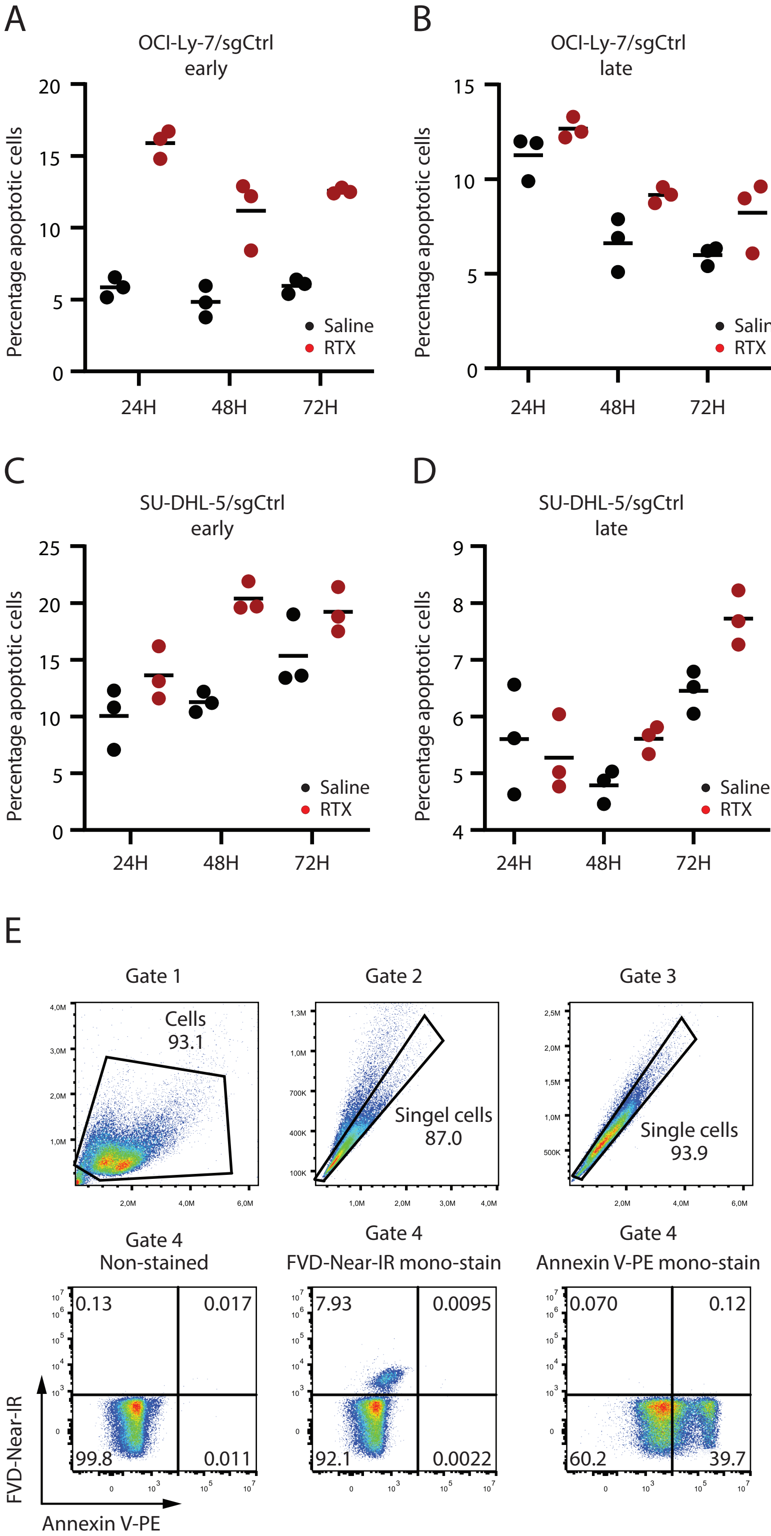

Supplemental figure S8

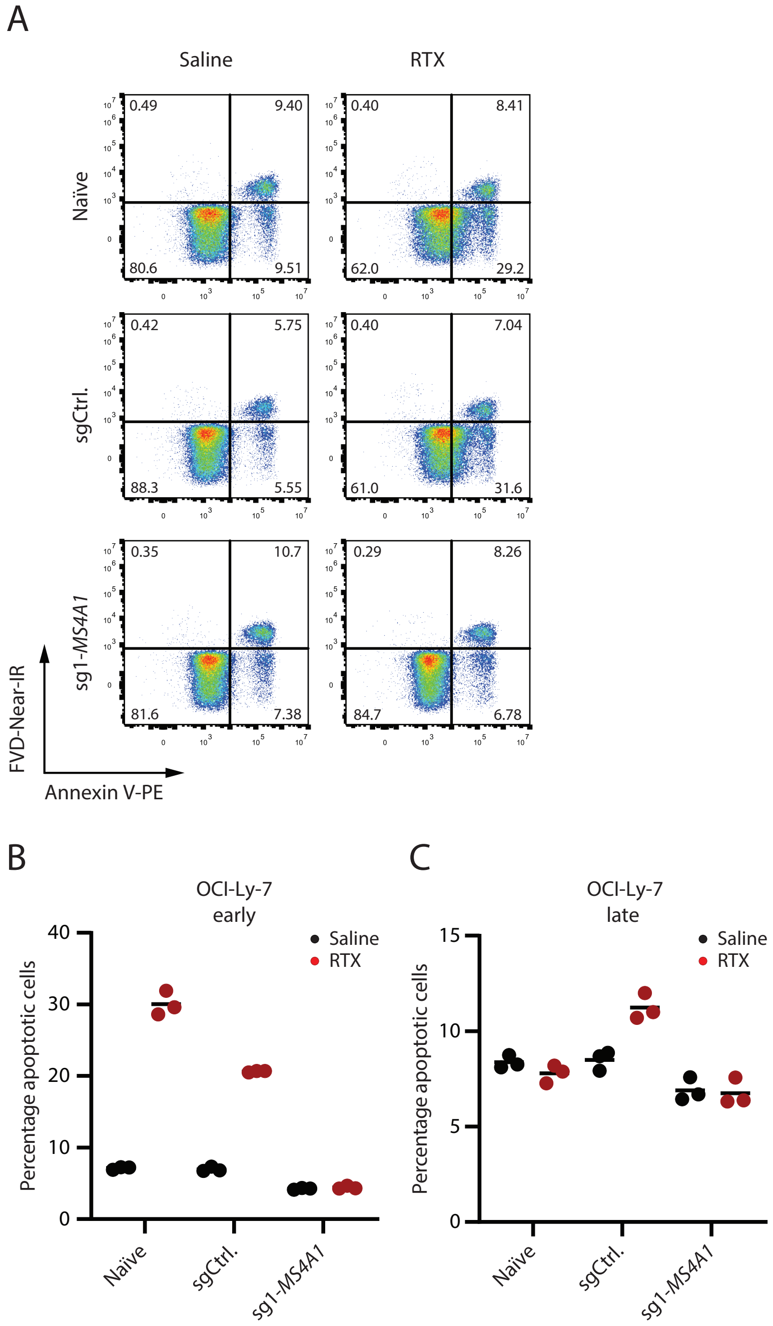

Supplemental figure S9

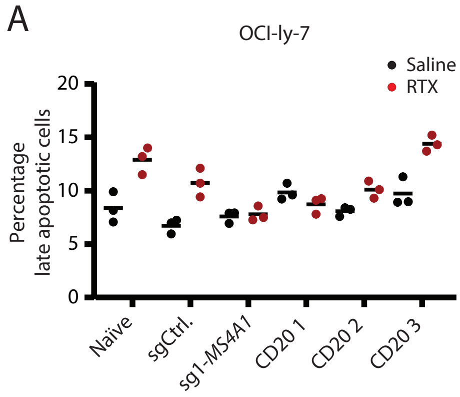

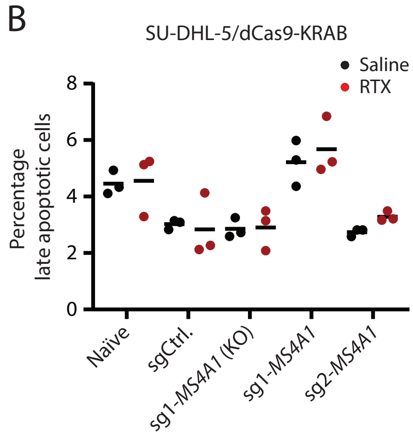

Supplemental figure S10

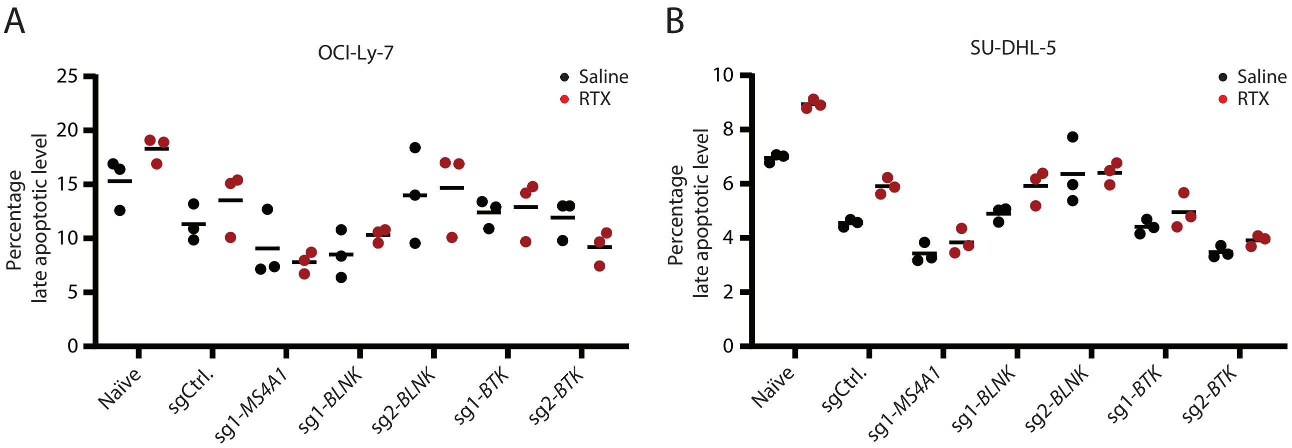

Supplemental figure S11

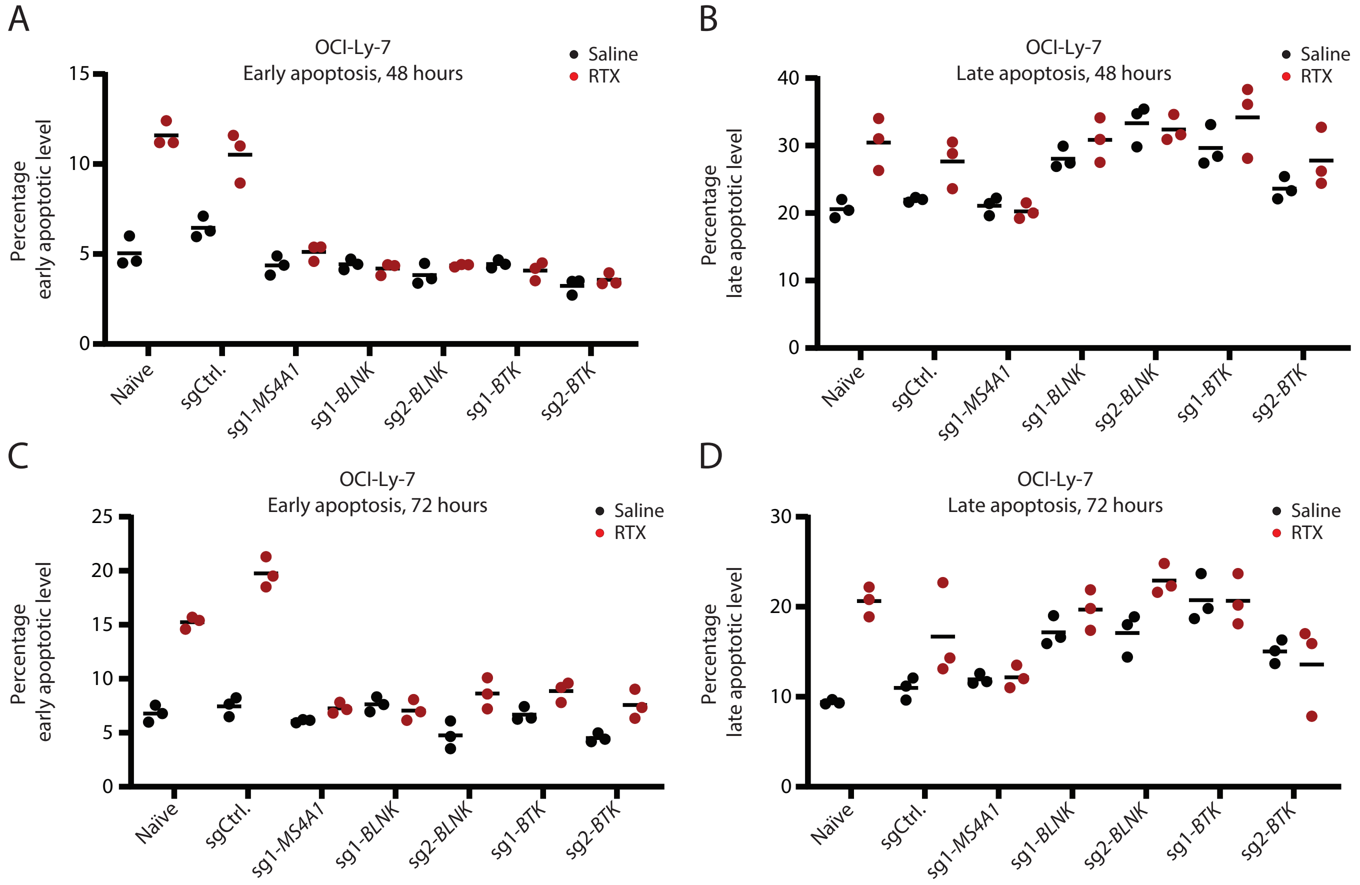

Uncropped western blot membranes

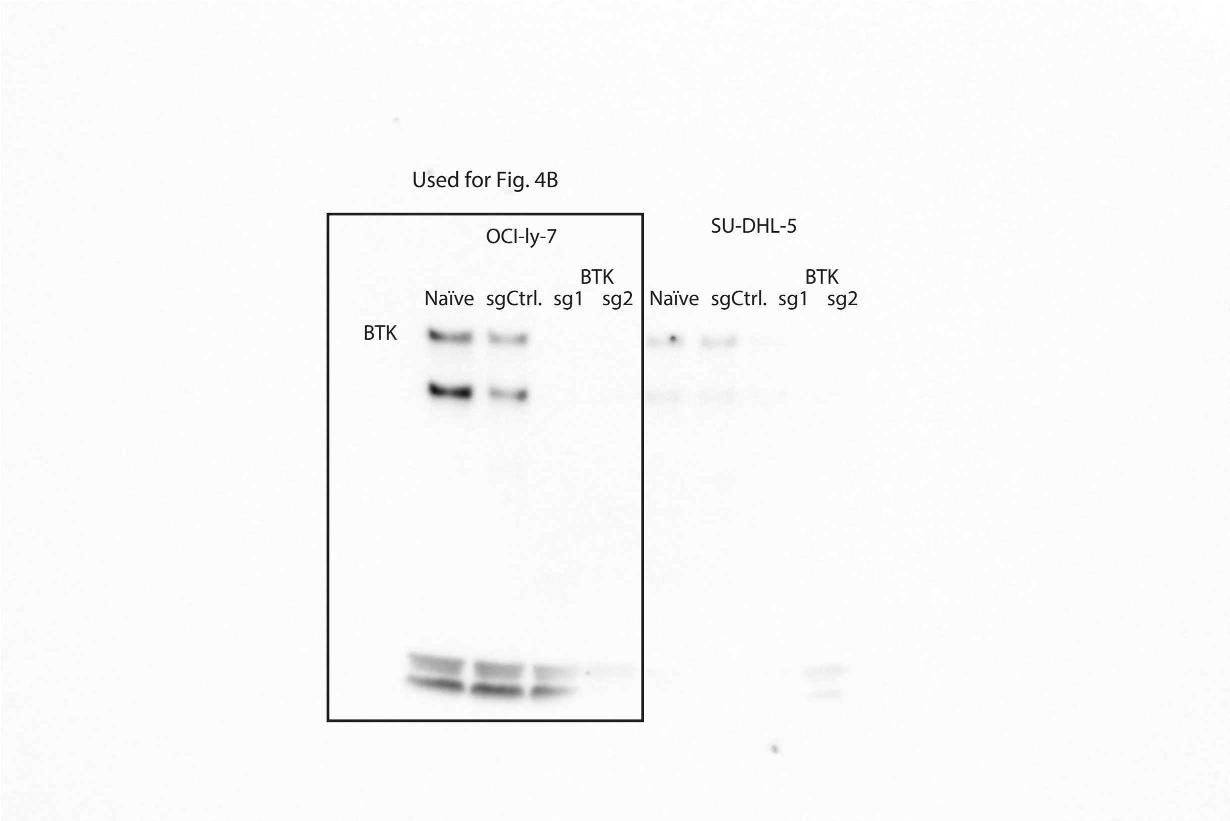

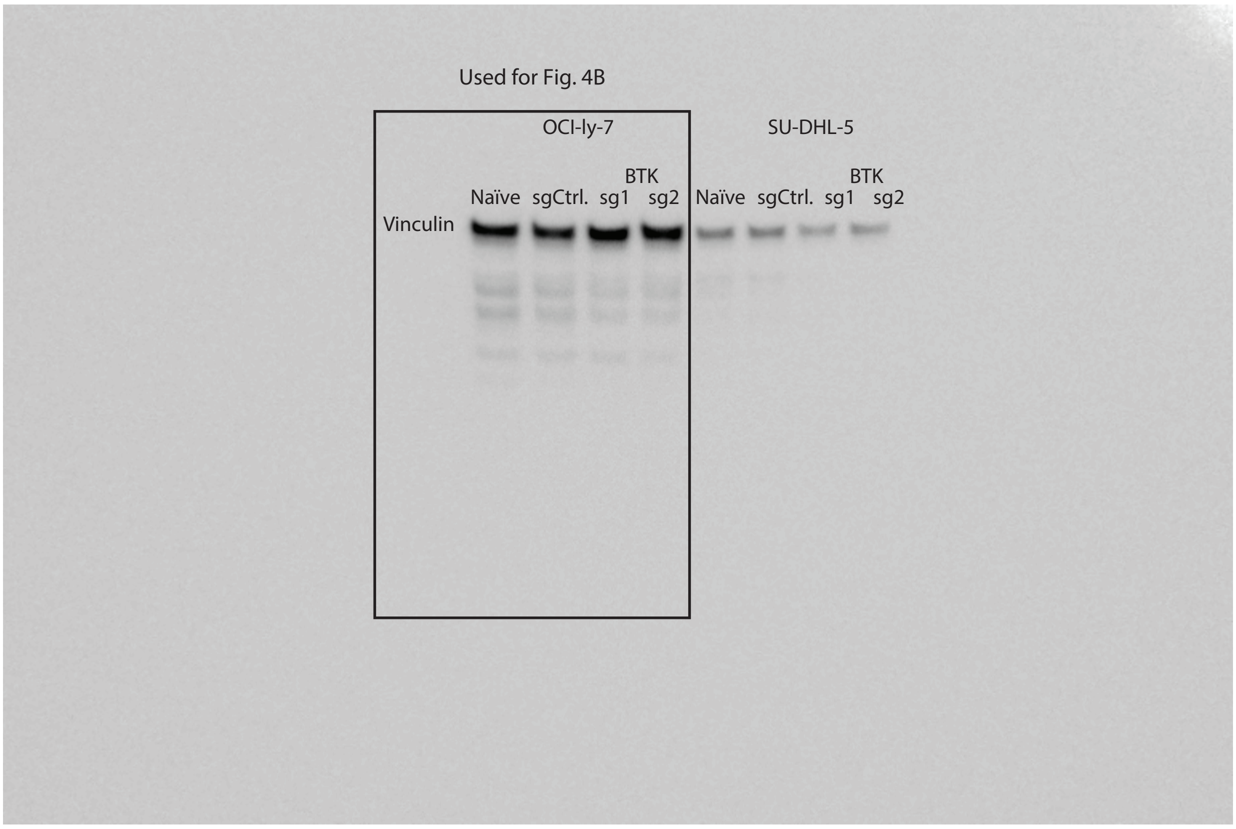

Uncropped western blot membranes

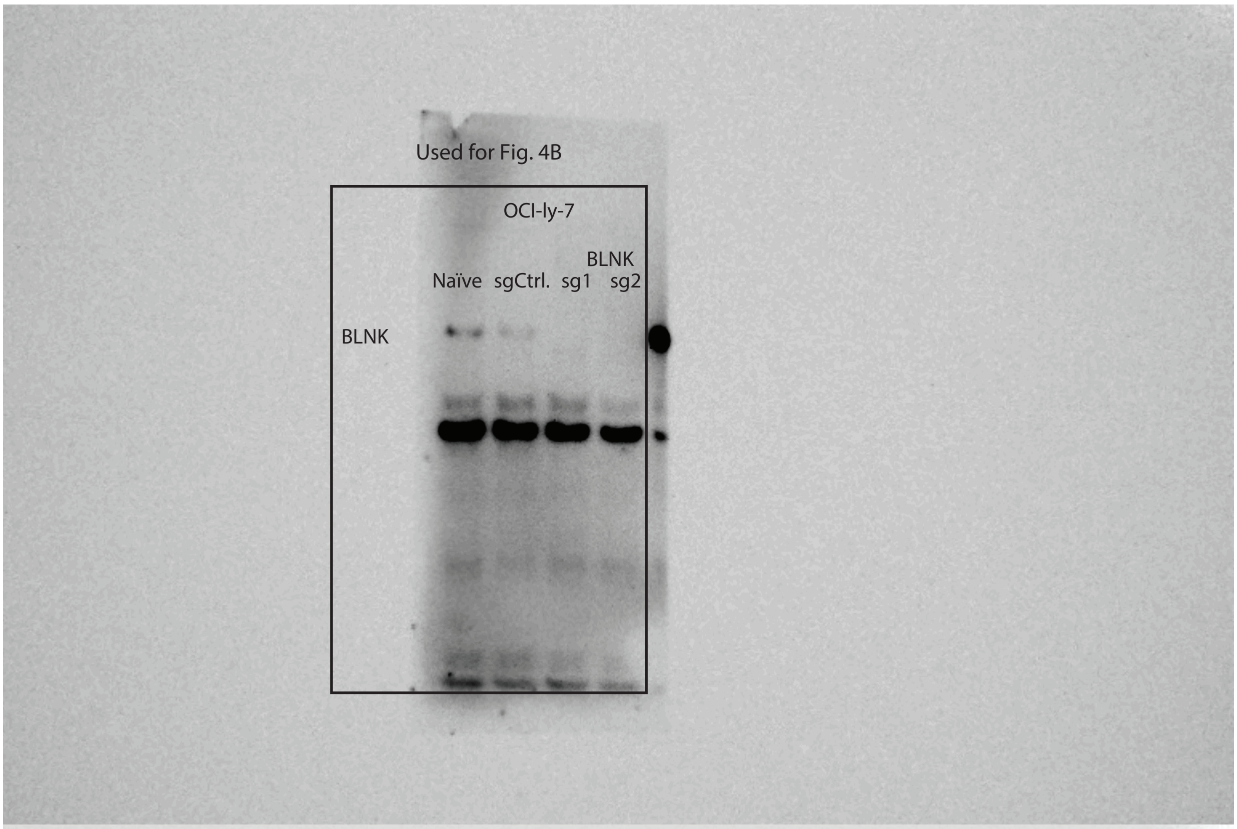

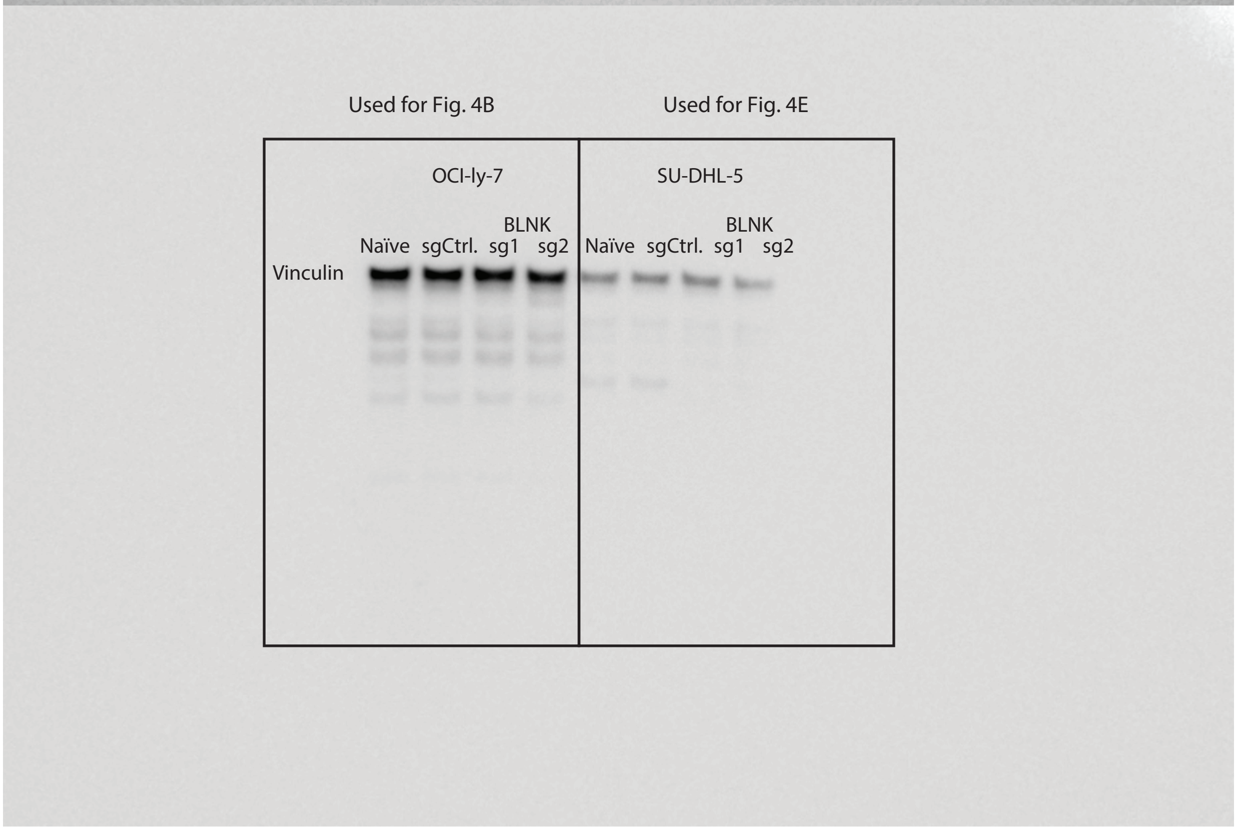

Uncropped western blot membranes

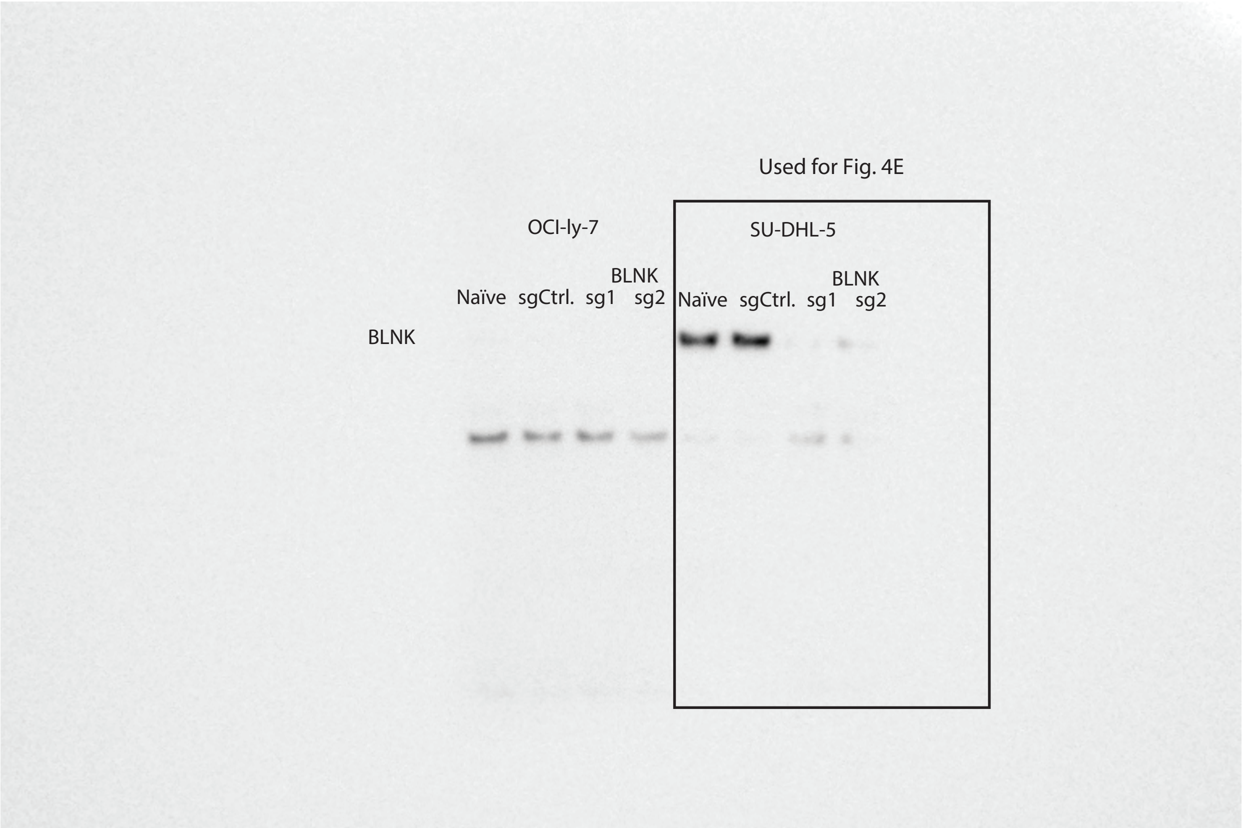

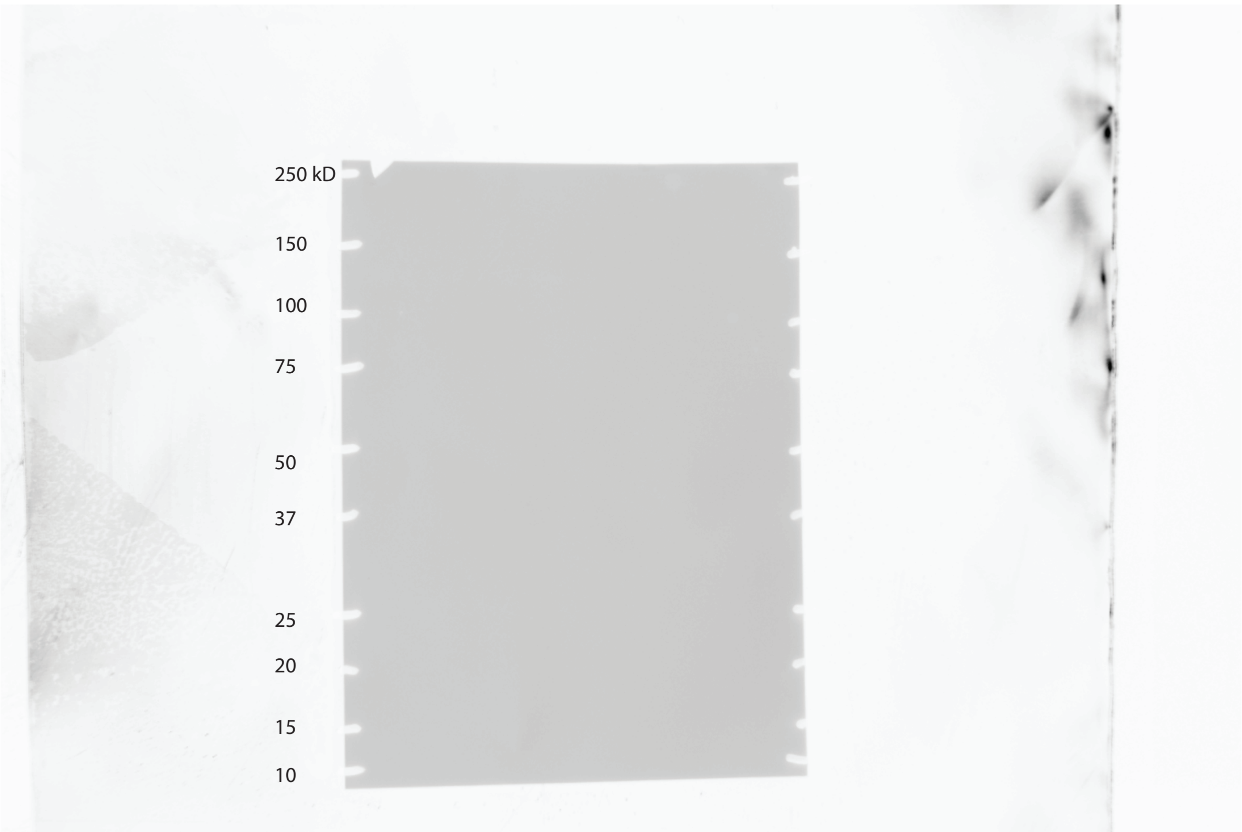

Uncropped western blot membranes

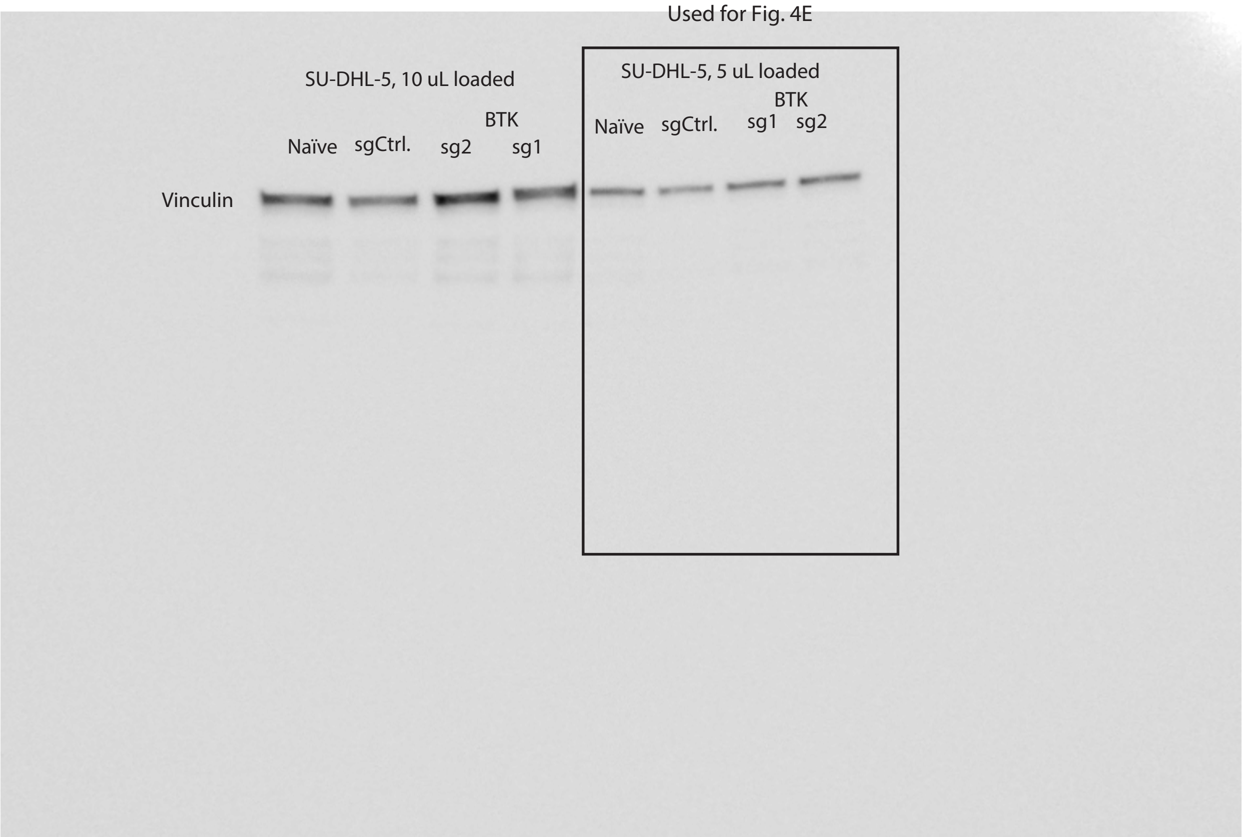

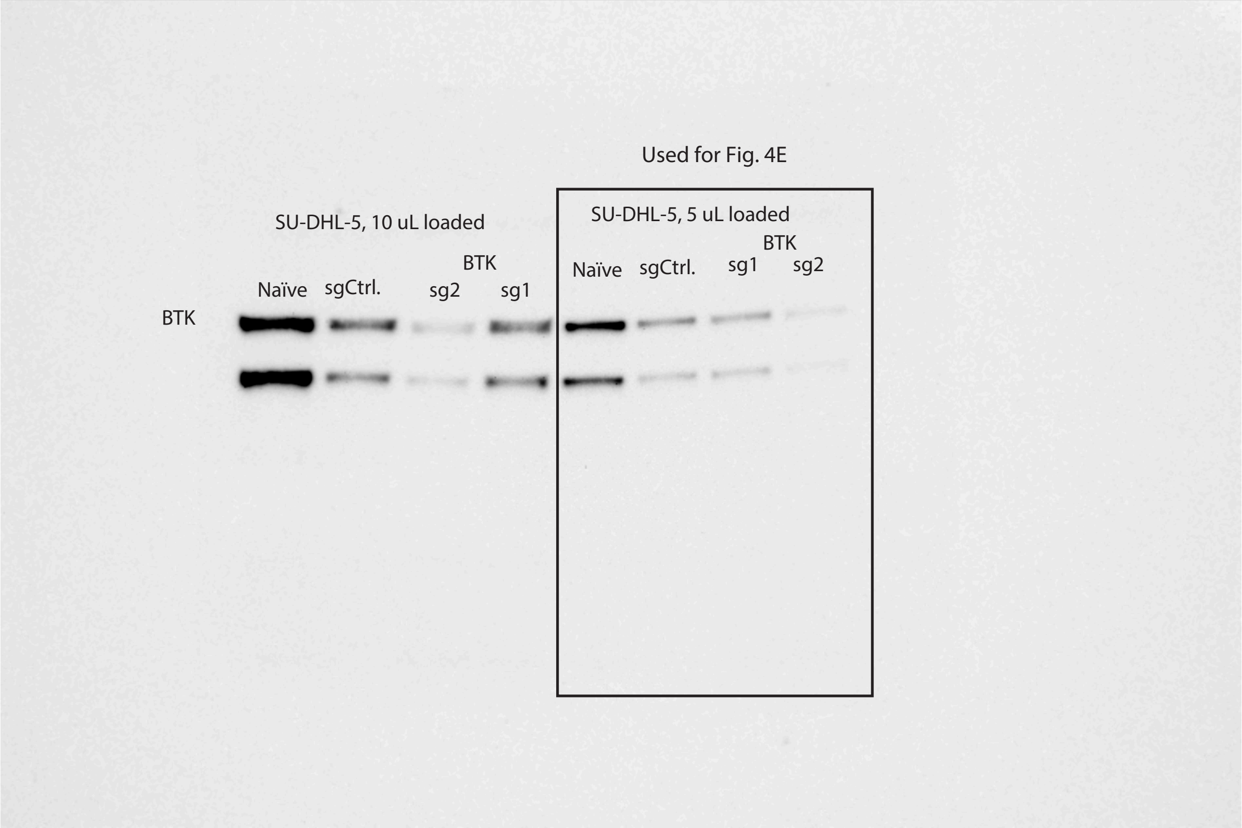

Uncropped western blot membranes

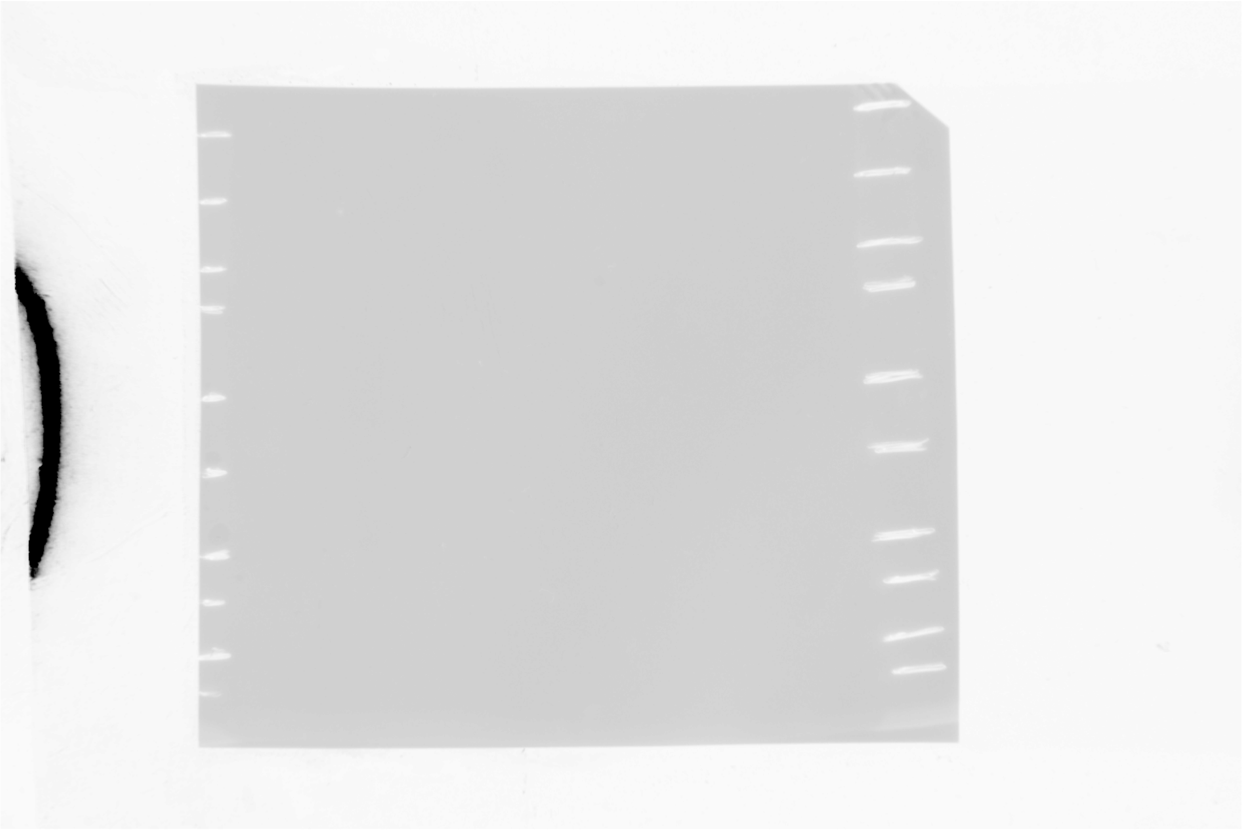

Supplement: Supplementary file 1 — Fig. S1. Efficient lentiviral transfer to malignant B‐cells at low MOI. (A) Schematic representation of vectors pCCL/PGK‐eGFP and pLentiCRISPRv2, the latter which was used to produce KO cell lines. (B) Transductional titers of LV/PGK‐eGFP and LV/CRISPRv2 in OCI‐Ly‐7 cells. Copy numbers following transduction were determined by qPCR; number of transducing units per milliliter is displayed. Three separate transductions were performed, and qPCR were performed in technical duplicates; shown is mean. (C) Median enhanced green fluorescent protein (eGFP) fluorescence in OCI‐Ly‐7 and SU‐DHL‐5 cells following transduction with different doses of LV/PGK‐eGFP. MOI estimates are based on qPCR. (D) Percentage of eGFP‐positive OCI‐Ly‐7 and SU‐DHL‐5 cells following transduction with different doses of LV/PGK‐eGFP. MOI estimates are based on qPCR; three separate samples from each population were prepared for flow. Mean is shown. Fig. S2. Lentiviral gene delivery to B‐cell lines does not affect CD20 surface levels. CD20 surface expression in OCI‐Ly‐7 (A) and SU‐DHL‐5 (B) populations transduced with different lentiviral constructs. Three transductions per dose were performed. Illustrated ratio represent median CD20 fluorescent intensity relative to naïve population; shown is mean. (C) Gating strategy for flow cytometric assessment of CD20 expression levels. Fig. S3. CRISPR library delivery and representation. (A) Transduction titer of lentiviral preparation used to deliver the Gecko v2 CRISPR library. Copy numbers following transduction were determined by qPCR; number of transducing units per milliliter is displayed. Three separate transductions were performed, and qPCR were performed in technical duplicates. Shown is mean. (B) Violin plots of sgRNA read counts across all samples; log2‐normalized read counts have been plotted. Fig. S4. Response to rituximab dependent on exposure time. Rituximab drug assay with non‐CDC conditions in OCI‐Ly‐7 cells expressing control sgRNA. Cells were [file MOL2-14-1978-s001.pdf]
